# Playing with Structural Parameters in the Design of Ruthenium(II)–*p*‑Cymene Complexes as Potential Antibacterial Agents

**DOI:** 10.1021/acs.inorgchem.5c03505

**Published:** 2025-11-19

**Authors:** Gina Elena Giacomazzo, Valentina Ceccherini, Valentina Vitali, Luca Conti, Francesca Vaccaro, Francesca Coscione, Elena Perrin, Marco Fondi, Lara Massai, Claudia Giorgi, Luigi Messori

**Affiliations:** † Department of Chemistry “Ugo Schiff”, 9300 University of Florence, via della Lastruccia 3, 50019 Sesto Fiorentino (FI), Italy; ‡ Department of Biology, University of Florence, via Madonna del Piano 6, 50019 Sesto Fiorentino (FI), Italy

## Abstract

A series of ruthenium­(II) arene complexes of the general formula [(η^6^-pCymene)­Ru­(L)­Cl]­PF_6_ (hereafter RACs) have been designed, synthesized, and characterized with a view to their possible use as antimicrobial agents. We specifically report on five structurally related RAC complexes, i.e., **Ru-pCy1–5,** incorporating bidentate polypyridyl ligands of different nature (**L1** = benzo­[i]­dipyrido­[3,2-a: 2′,3′-c]­phenazine, **L2** = 4,7-diphenyl-1,10-phenanthroline, **L3** = 2,2′-biquinoline, **L4** = 2,2′-bipyridine-4,4′-diylbis­(morpholinomethanone), **L5** = (2,2′-bipyridine-4,4′-diylbis­(morpholinomethylene)). The complexes **Ru-pCy1–5**, which showed substantial inertness when dissolved in aqueous media, were investigated by ESI-MS for their ability to interact with two representative model proteins, i.e., SOD and RNase A. Interestingly, these complexes, with the sole exception of **Ru-pCy3**, tend to form stable adducts with the two proteins upon release of the chloride ligand, in agreement with previous observations on similar Ru compounds. The antimicrobial properties of these compounds were then tested against Gram-positive bacterium *Bacillus subtilis* and Gram-negative bacterium *Burkholderia cenocepacia*. The observed antibacterial effects were then tentatively correlated with the structural features of each complex.

## Graphical abstract

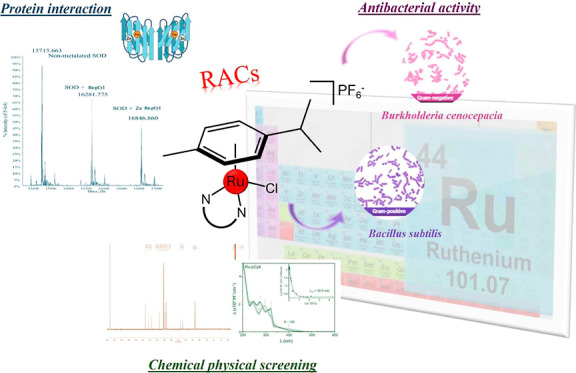

## Introduction

Tackling antimicrobial resistance (AMR) is a global priority. Beyond promoting a more responsible use of antibiotics, there is an urgent need to develop novel and effective antimicrobials, preferably based on a new class of compounds, rather than on analogues of known scaffolds.^1^

In this context, the contribution of inorganic chemistry is attracting increasing attention, due to the unique redox, coordination, and structural properties of transition metal-based antimicrobials, which may offer key advantages over their purely organic counterparts.^2–4^ Among inorganic compounds, ruthenium polypyridyl complexes (RPCs) are showing promise as antimicrobials,^5–7^ extending their well-established use as anticancer agents.^8–10^

An even greater potential may lie in the closely related class of ruthenium­(II) arene complexes (RACs), with general formula [(η^6^-arene)­Ru­(L)­X]­PF_6_ (RACs). Indeed, despite being widely studied as “non-platinum” anticancer agents,^11–14^ the potential of these versatile “piano-stool” complexes as antibacterial alternatives has been far less explored.^15,16^

Among the main reasons behind the role of RACs as antibacterial agents, there is their versatile pseudo-octahedral geometry, where the three key components, namely, the η^6^-arene ligand, the bidentate ligand (L), and the monodentate ligand (X), can be easily modified to precisely modulate the chemical–physical properties of the resulting compound. This, in turn, can be exploited to generate libraries of metal complexes with different structures, useful for gaining valuable insights into structure-activity relationships (SARs).

The role of the η^6^-arene moiety for the biological and pharmaceutical application of RACs is mainly related to its strong σ-donor ability, which stabilizes the ruthenium ion in its +2 oxidation state and provides a hydrophobic surface that can enhance cellular uptake by increasing passive diffusion across cell membranes.^17,18^ Aliphatic groups attached to the aryl moiety can further enhance cytotoxicity by increasing the hydrophobic interaction with DNA and/or proteins.^19–22^ The nature of the monodentate ligand (X) is another crucial factor, as labile X ligands can be replaced by a water molecule to produce unsaturated Ru­(II) aqua species capable of strongly interacting with important biological targets such as proteins and nucleic acids. An additional control over the chemical–physical properties and, ideally, the biological behavior of the resulting drug candidates can be achieved by varying the nature of the bidentate (L) ligand, as it has been previously shown for RACs containing, for example, α-diimine,^19,22–28^ thiosemicarbazone,^29,30^ hydrazone,^31^ and thiolate^12,32,33^ bidentate ligands.

Taking into account these aspects, and in an attempt to reduce the gap between the extensive anticancer studies and the still limited use of these compounds as antibacterial agents, in this work, we report on a series of five monocationic complexes, of the general formula [(η^6^-*p*-Cymene)­Ru­(L)­Cl]^+^. These complexes were rationally designed using (i) *p*-cymene as the η^6^-arene ligand, with the methyl and isopropyl groups aimed at enhancing hydrophobic interactions and thereby increasing affinity toward biological targets;^22^ (ii) a labile chloride X ligand to facilitate the Ru–Cl cleavage, providing unsaturated species that can readily interact with biological targets,^24^ and (iii) polypyridyl ligands of different nature as l-bidentate chelates, inspired by previous ruthenium­(II) arene-based antitumor^23,27,34^ and cancer diagnostic agents.^19,26^

Our series included the literature compounds **Ru-pCy1**,^26,35^ **Ru-pCy2**,^36^ and **Ru-pCy3**,^37,38^ which contain benzo­[i]­dipyrido­[3,2-*a*:2′,3′-*c*]­phenazine (dppn, **L1**), 4,7-diphenyl-1,10-phenanthroline (DIP, **L2**), and 2,2′-biquinoline (biq, **L3**), respectively, as L ligands. These ligands differ in the degree of π-extension and could therefore potentially influence key factors such as cell membrane penetration^1,34^ or Ru–Cl hydrolysis.^37^ Additionally, inspired by the central role that morpholine plays in a wide range of pharmaceutical agents, including the popular antimicrobial linezolid,^36^ and by the recent demonstration of its potential when incorporated into Ru­(II) polypyridyl complexes as effective metalloantibiotics with multiple antibacterial mechanisms,^39^ we synthesized and included in this study two novel complexes, **Ru-pCy4** and **Ru-pCy5**. In these complexes, two morpholine moieties are linked to the Ru­(II)-coordinated bipyridyl units via either an amide (**L4** in **Ru-pCy4**) or a methylene (**L5** in **Ru-pCy5**) spacer (Scheme 1).

**Scheme 1:**
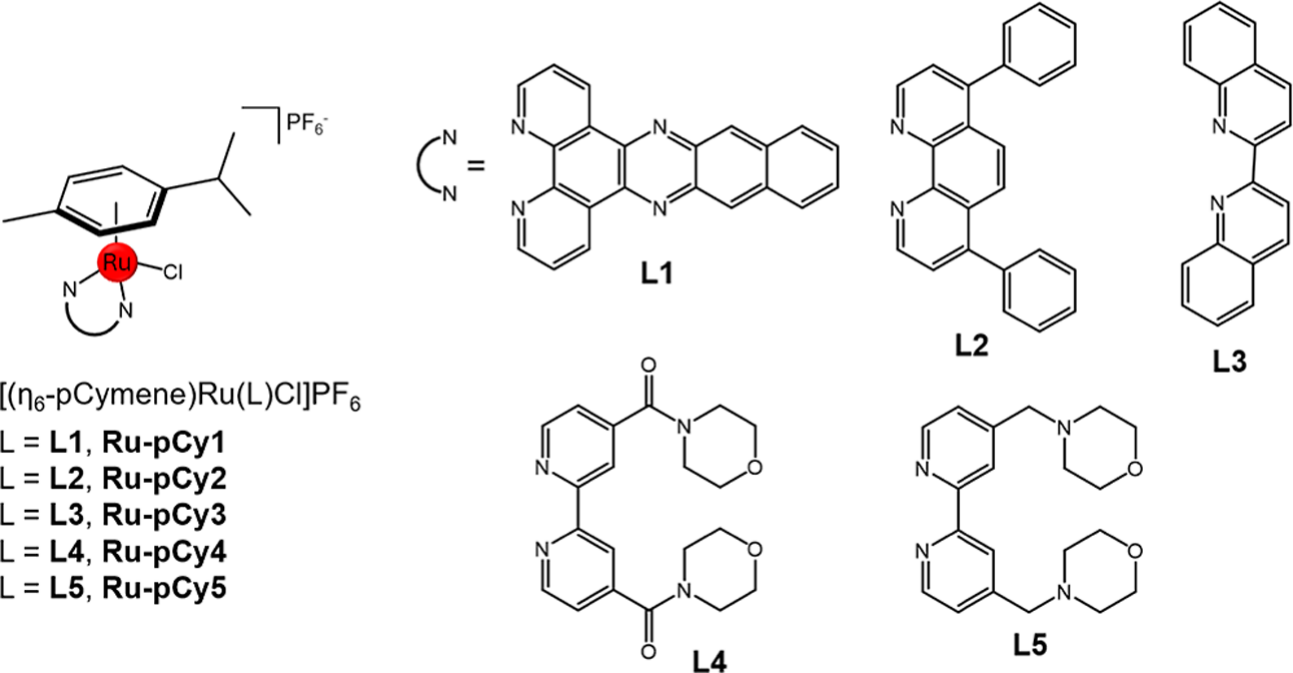
**Ru-pCy1-5** RAC Complexes with Their Bidentate Polypyridyl Ligands **L1–L5**

Following the synthesis of **Ru-pCy1–5**, carried out by modifying literature protocols,^19,26,35,37^ we conducted a comparative study of their physicochemical properties relevant to pharmaceutical use. We also investigated their ability to interact with proteins as potential biological targets using electrospray ionization mass spectrometry (ESI-MS) and evaluated their antibacterial activity preliminarily.

The ultimate aim of this work is to advance our understanding of the antibacterial potential of this class of compounds, thereby providing a rationale for the design of new antimicrobial alternatives based on RACs. It also seeks to narrow the gap between the well-documented anticancer behavior and the less explored antimicrobial use of this class of compounds.

## Results and Discussion

### Synthesis and Characterization of RAC Complexes

The synthesis of ruthenium complexes **Ru-pCy1–5** was carried out according to the synthetic strategy shown in Scheme 2, after the preparation of bidentate ligands **L1**–**L5**.

**Scheme 2:**
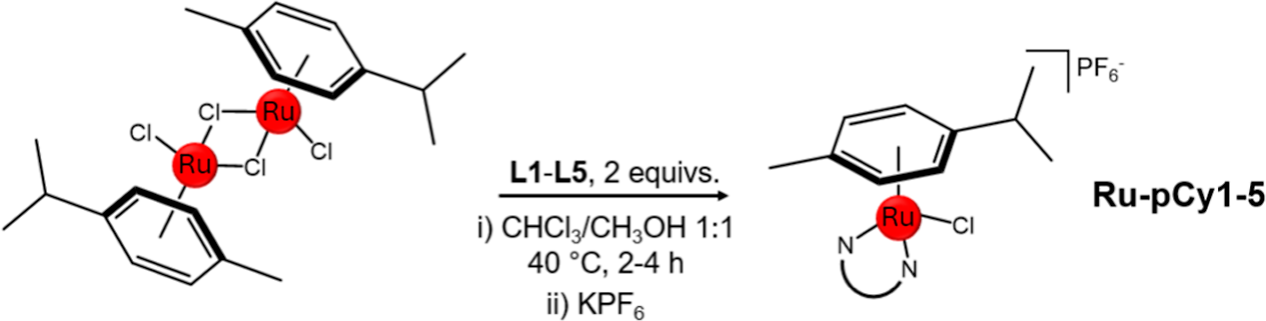
Schematic Representation of the Synthetic Route Followed for the Synthesis of **Ru-pCy1–5**

**L1** was prepared according to the literature,^40^ while **L2** and **L3** were purchased as commercially available compounds. The synthesis of **L4** (2,2′-bipyridine-4,4′-diylbis­(morpholinomethanone)), with two morpholine moieties attached to a 4,4′-dicarbonyl-2,2′-bipyridine moiety via an amide bond, has been recently described by our group.^41^ The novel polypyridyl ligand, **L5** (2,2′-bipyridine-4,4′-diylbis­(morpholinomethylene)), was also prepared to exploit the different functionalization of the morpholine pendant arms. The synthesis of this ligand, in which the morpholine groups are linked as tertiary ammines to methylene bridges at the 4,4′ position of the 2,2′-bipyridine, was carried out by nucleophilic substitution of the bromine derivative 4,4′-bis­(bromomethyl)-2,2′-bipyridine with morpholine in anhydrous acetonitrile using potassium carbonate as the base.

The synthesis of **Ru-pCy1–5** was achieved by slight modifications of methods reported in the literature for **Ru-pCy1–3** (Scheme 2). Briefly, 2 mol equiv of the selected ligand (**L1–5**) was allowed to react with a dichloro­(*p*-cymene)­ruthenium­(II) dimer in a chloroform–methanol (1:1) mixture at 40 °C. Addition of aqueous or methanolic solution of KPF_6_ resulted in the precipitation of complexes as hexafluorophosphate salts. RACs were then purified by flash chromatography on silica gel, giving **Ru-pCy1–5** in 67–92% yield.

All complexes were fully characterized by ^1^H, ^13^C, and COSY and HSQC NMR spectroscopy and high-resolution mass spectrometry (HR-MS) analysis (see Supporting Information, Figures S5–S25). In the ^1^H NMR spectra of all compounds, both the *p*-cymene and polypyridyl ligands show a single set of protons, indicating the retention of the ligand symmetry after coordination to ruthenium. Identity of the compounds was further confirmed by the characteristic molecular ions [M-PF_6_]^+^ in all HR-MS spectra of the complexes.

The electronic absorption and fluorescence spectra of complexes **Ru-pCy1-5** were recorded in acetonitrile and water; the obtained spectra are showed in Figure 1, whereas their molar extinction coefficients (ε) at different absorption maxima (λ_max_) in both solvents are listed in Table 1. All complexes, except for **Ru-pCy1**, exhibit intense absorption bands in the 220–320 nm region, ascribable to π → π* transitions, together with a low-energy absorption in the 350–450 nm range, characteristic of the metal-to-ligand charge transfer (MLCT) transition. In case of **Ru-pCy1**, the intense absorption bands at 323, 400, and 422 nm, ascribable to the **L1** π → π* transition,^41,42^ mask the MLCT band. Complexes generally show only moderate emission, with **Ru-pCy1** and **Ru-pCy5** exhibiting the highest luminescence signal in acetonitrile upon excitation at 400 and 230 nm, respectively. The emissive behavior of **Ru-pCy1** is consistent with the beneficial role imparted by the π-extended dppn ligand in enhancing radiative decay, as previously observed for other Ru­(II)–arene complexes.^43^ The emission of **Ru-pCy5**, particularly considering the nonemissive character of its closely related analogue **Ru-pCy4**, likely arises from the different nature of the linker between the morpholine derivatives and the bipyridine unit. In the former case indeed, the intramolecular quenching role often associated with amide and amide-like functionalities may promote nonradiative deactivation through electron-withdrawing and vibrational effects.^44,45^

**Figure 1:**
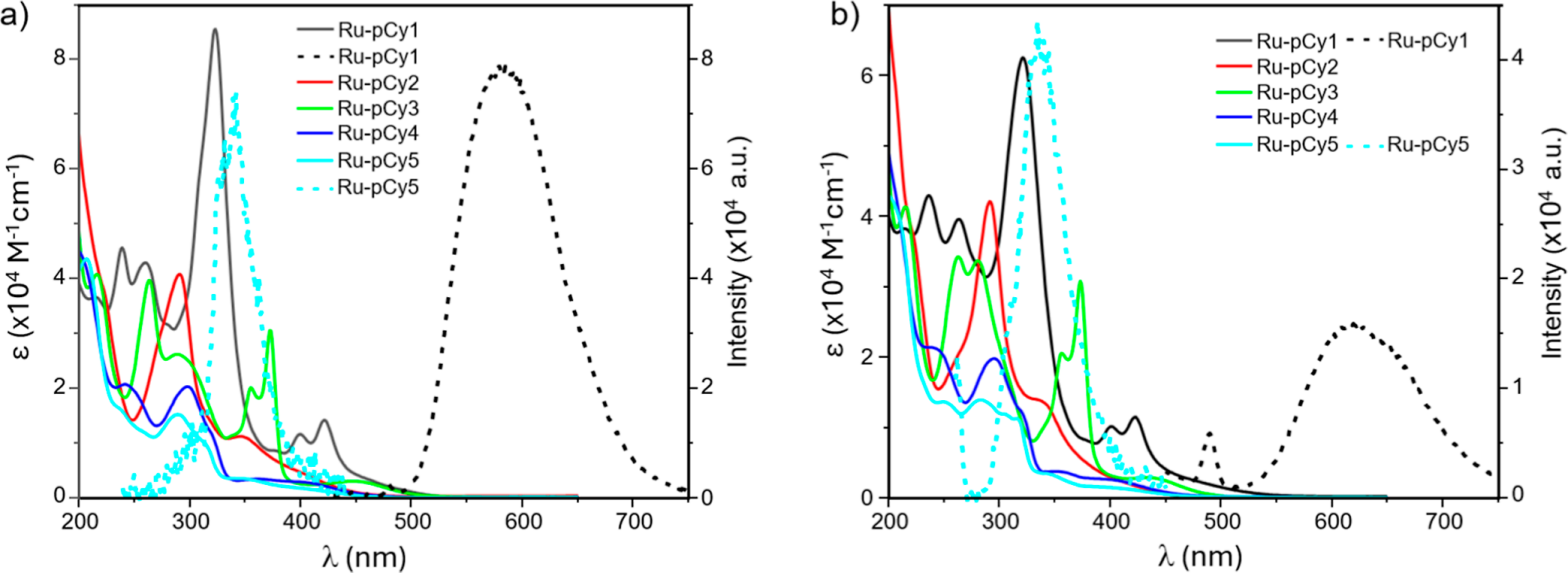
Electronic absorption and fluorescence spectra (straight and dotted lines, respectively) of ruthenium complexes **Ru-pCy1–5** (10 μM) recorded in acetonitrile (a) and water (b). For emission spectra, **Ru-pCy1** and **Ru-pCy5** were excited at 400 and 230 nm, respectively, in acetonitrile and at 420 and 240 nm in water, respectively. No emission was observed for the other metal complexes of the series.

**Table 1:** Absorption and Fluorescence Maxima (λ_max_/λ_em_) of **Ru-pCy1–5** in Acetonitrile and Water and Their Water–Octanol Partition Coefficients (Log *P*_pH7.4_)

	λ_max_/nm (ε × 10^3^ M^–1^ cm^–1^) in CH_3_CN	λ_max_/nm (ε×10^3^ M^–1^ cm^–1^) in H_2_O	λ_em_ in CH_3_CN	λ_em_ in H_2_O	Log *P*_pH7.4_	*k*_water_ (× 10^–4^ s^–1^)	*t*_1/2_ (min)
**Ru-pCy1**	422 (14.0), 400 (11.5), 323 (85.5), 260 (42.8), 240 (45.3)	423 (11.5), 402 (10.2), 322 (62.5), 264 (39.6), 237 (43.0)	585 nm (λ_exc_ 400 nm)	624 (λ_exc_ 420 nm)	0.80 ± 0.30	2.5 ± 0.1	46.5 ± 2.0
**Ru-pCy2**	347 (11.1), 292 (40.6)	342 (13.3), 292 (42.1)	-	-	0.91 ± 0.30	4.5 ± 0.2	25.7 ± 1.2
**Ru-pCy3**	456 (2.82), 373 (30.4), 357 (19.6), 290 (26.1), 265 (39.1)	442 (2.82), 372 (30.8), 357 (20.6), 280 (33.6), 265 (33.9)	-	-	0.57 ± 0.19	-	-
**Ru-pCy4**	407 (26.9), 299 (20.2)	403 (2.61), 358 (3.72), 296 (19.8), 245 (21.1)	-	-	–0.66 ± 0.23	3.7 ± 0.4	30.9 ± 3.4
**Ru-pCy5**	399 (17.9), 290 (15.1)	406 (1.49), 345 (3.48), 317 (11.3), 283 (14.0), 251 (13.7)	340 nm (λ _exc_ 230 nm)	337 nm (λ _exc_ 240 nm)	–0.63 ± 0.30	5.4 ± 0.4	21.1 ± 1.8

### RAC Complexes: Stability in Solution and Log *P*_pH7.4_ Values

To gain insights into the suitability of RACs for biological purposes, their stability in aqueous solution was investigated before studying their interactions with representative proteins (SOD, RNase A, and CytC) by ESI-MS analysis (vide infra). Measurements were performed under strictly controlled conditions (temperature, pH, and complex concentration), namely, at a concentration of 1 × 10^–5^ M in CH_3_CN/PBS solution (CH_3_CN 1% v/v) at room temperature, over a total of 72 h. The resulting time-dependent absorption profiles are reported in Figure S26 (Supporting Information). As shown, the consistent absorption profiles over the investigated time frame indicated a remarkable stability of all ruthenium complexes, with the only exception of **Ru-pCy1**, which showed a moderate tendency to precipitate at the longest time points, likely due to the aggregation tendency imparted by the dppn units in an aqueous medium.^46^

Additionally, the lipophilic/hydrophilic character of **Ru-pCy1–5** was measured. This is of particular interest given the structural features of the complexes, such as their monocationic nature and the presence of the *p*-cymene moiety, which can enhance lipophilicity,^47^ as well as the variable polarity and hydrogen-bonding capacities of their bidentate ligands (particularly of **Ru-pCy4 and 5**), which may further influence solubility and partitioning behavior. An optimal balance between lipophilicity, favoring cell membrane diffusion and interaction with biological targets, and adequate solubility in physiological media, is indeed an issue of undoubted importance.^48^

The study was performed by using the shake-flask method, obtaining the water–octanol partition coefficients of complexes in PBS buffer at pH = 7.4 (Log *P*_pH7.4_), as listed in Table 1.

As shown, the obtained Log *P*_pH7.4_ values range from −0.66 to +0.91, with positive and negative values found for **Ru-pCy1–3** and **Ru-pCy4 and 5**, denoting an imbalance between lipophilic and hydrophilic character. The extent of the distribution coefficients of **Ru-pCy1–3** roughly mirrors the degree of π-extension of polypyridyl chelates across the series, whereas, on the other side, the lower (negative) values achieved for **Ru-pCy4** and **Ru-pCy5** can be easily explained with the enhancement of hydrophilicity imparted by their morpholine residues.

This further highlights that the overall Ru­(II)–arene architecture, the aromaticity of L ligands, and the morpholine groups can be strategically combined to tune the lipophilic/hydrophilic balance of the resulting metal complexes.

### Aquation of RAC Complexes

In view of the well-known lability of the Ru­(II)–Cl bond in RACs and considering its relevance for their biological mechanism of action,^22^ the aquation process of all systems was investigated. This was performed according to literature methods^49^ spectrophotometrically, by registering the UV–vis spectra of freshly prepared aqueous solutions of complexes at neutral pH over different times, up to 12 h, at 298 K. Results for **Ru-pCy1** are shown in Figure 2, while those for the other complexes are reported in Figures S27–S28 Supporting Information. As shown, **Ru-pCy1** promptly displayed significant variations over time, especially in the MLCT region (400–430 nm), which exhibits a progressive blue-shift, consistent with the replacement of the chloride ligand with the stronger ligand-field water molecule.^26^ The temporal evolution to the aquo-species is also further supported by comparison of spectra collected at increasing time frames and the absorption profile of the aquo species, registered by adding AgNO_3_ to the metal complex (Figure 2b). A similar behavior was also observed for the other compounds, confirming their capability to readily lose the chloride ligand. Interestingly, the only exception was found for **Ru-pCy3**, which did not show appreciable variations within the time frame investigated (Figure S27). Hydrolysis was not observed during the stability experiments (Figure S26), likely being suppressed by the high NaCl concentration in the PBS buffer media (140 mM).

**Figure 2:**
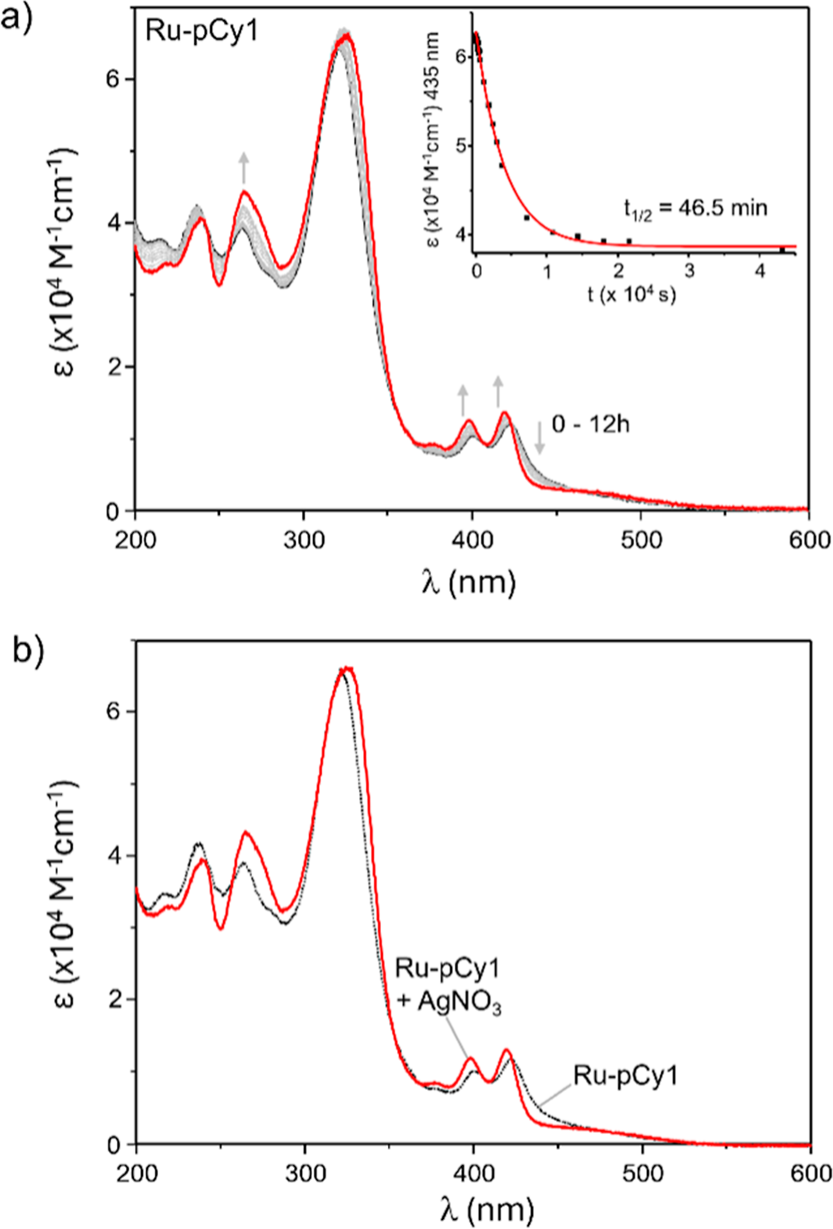
Ru–Cl hydrolysis for **Ru-pCy1**, followed by UV–vis measurements in aqueous solutions at different times (up to 12 h). In (a) are reported the obtained time-dependent spectra, the inset showing the variation at a maximum at 435 nm as a function of the time. In (b) are compared the absorption spectra without and in the presence of 1 equiv of AgNO_3_ ([**Ru-pCy1**] = 10 μM, pH 7, 298 K).

The aquation rate constants (*k*_water_) and the corresponding half-lives (*t*_1/2_) were determined by the time-dependent absorbance changes at the MLCT maxima according to a pseudo-first-order kinetic model. The resulting values, reported in Table 1, range between 2.48 and 5.4 × 10^–4^ s^–1^. The fastest processes were observed for **Ru-pCy5** (*t*_1/2_ ∼21 min) and confirm the tendency of all the series to readily undergo chloride substitution in aqueous media, except for the less active **Ru-pCy3**. These values are also in line with those reported in the literature for analogous Ru­(II)–arene complexes;^50^ minor deviations were observed for **Ru-pCy2**,^50^ likely ascribable to the slightly different experimental conditions employed (ionic strength, temperature).

The inferior activity of **Ru-pCy3** was further corroborated by ESI-MS analysis. As shown in Figure S30, after 24 h of equilibration in aqueous solution, the spectrum largely retained the molecular peak of the starting complex [Ru­(*p*-cymene)­(**L3**)­Cl]^+^ ([M]^+^ *m*/*z* = 527.0840) showing only a small percentage of the doubly charged species from chloride loss, [Ru­(*p*-cymene)­(**L3**)]^2+^ ([M]^2+^ *m*/*z* = 246.0558). This was in contrast to the results obtained under the same conditions for **Ru-pCy1**, as witnessed by the peak at *m*/*z* = 284.0621, which emerged following incubation in aqueous solution and corresponding to the species ([Ru­(*p*-cymene)­(**L1**)]^2+^).

The reduced lability of the chloride ligand in **Ru-pCy3** likely accounts for the lack of interaction with target proteins observed by ESI-MS analysis (vide infra) and further suggests that variations in electronic and stereoelectronic features arising from the specific molecular design of these complexes may play a key role in modulating chloride substitution.

Lastly, the tendency of the coordinated water ligand to undergo deprotonation was also considered. This has been investigated for the lead compound **Ru-pCy1**, by recording the UV–vis spectra of batchwise aqueous solutions of the complex at different pH values and monitoring the changes in the MLCT region (Figure S29). Fitting the variation in absorbance maxima yielded a p*K*_a_ value of 8.3 ± 0.1, indicating that the doubly charged [Ru­(*p*-cymene)­(**L1**)­(H_2_O)]^2+^ species predominates at neutral pH.

### ESI-MS Interaction Studies of RACs with Representative Proteins

To gain a deeper insight into the possible mode of interaction of RAC complexes with their likely biomolecular targets, we first analyzed, by ESI-MS measurements, their reactions with a few “model proteins”, often used by our group for this kind of investigations, i.e., ribonuclease A (RNase A), superoxide dismutase (SOD), and cytochrome c (CytC). ESI-MS analysis, performed according to established protocols,^51,52^ is indeed an excellent method to investigate and characterize metallodrug–protein interactions at the molecular level.

RNase A (124 residues, ∼13.7 kDa), CytC (104 residues, ∼12.6 kDa), and SOD (153 residues for subunit, ∼15.7 kDa) are relatively small proteins that represent classic model systems for the analysis of the interactions between proteins and metal compounds, since they are extremely stable and amenable to large-scale purification. The extensive characterization carried out in numerous previous studies also provides a robust body of existing data for comparison and validation. A fact further supported by previous investigations conducted by some of us revealed favorable affinity for both CytC and RNase A of similar heterometal (Ru–Au) complexes with the *p*-cymene ligand.^53^ Indeed, ESI-MS spectra recorded on protein samples after incubation with the individual RAC complexes can provide valuable insights on adduct formation, metal–protein stoichiometry, and the nature of protein-bound metal fragments.

Accordingly, ESI-MS spectra of RNase A, SOD, and CytC incubated for 24 h with a 3-fold molar excess of each of the five different ruthenium complexes were recorded. Interestingly, no adduct formation with the five RAC complexes was observed in the case of CytC; this led us to limit our study to RNase A and SOD. Moreover, among all complexes, **Ru-pCy3** did not interact with any of the three proteins investigated. As speculated above, this lack of interaction may be related to the inferior lability of the Ru­(II)–Cl bond found for this complex, which could prevent binding to the protein. This could be exacerbated by the steric hindrance of **L3**.

A consistent reactivity profile was observed for all the other RACs, namely, **Ru-pCy1, 2, 4, and 5**, with either SOD or RNase A, as indicated by the deconvoluted ESI-MS spectra obtained upon reacting with SOD or RNase A and synoptically reported in Figures 3 and S31, respectively.

**Figure 3:**
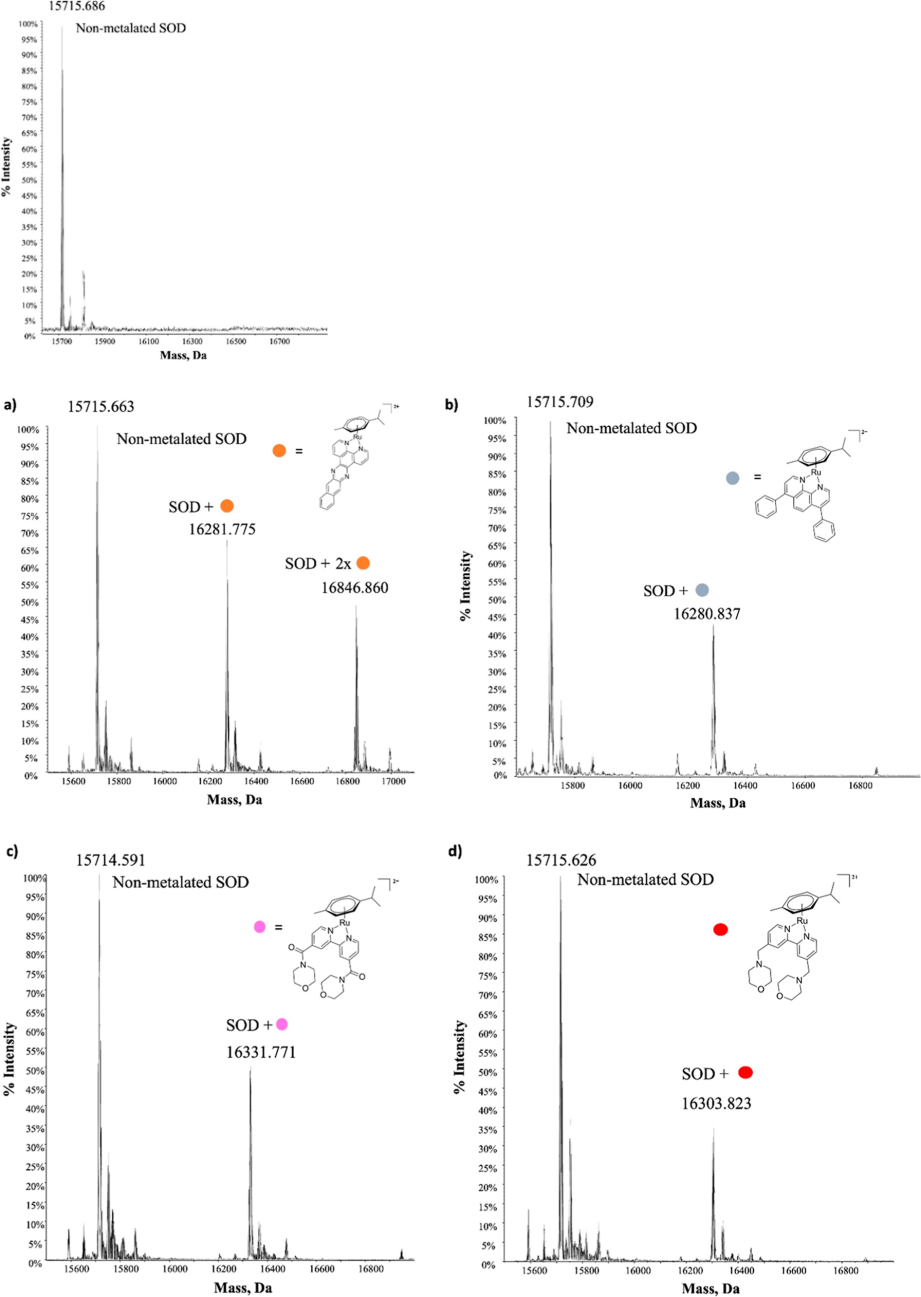
Deconvoluted ESI-Q-TOF spectra of nonmetalated SOD and the adducts with **Ru-pCy1** (a), **Ru-pCy2** (b), **Ru-pCy4** (c), and **Ru-pCy5** (d) complexes in a 1:3 protein to ruthenium ratio at 24 h.

In all cases, the metal frameworks interacting with proteins lack the original coordinated chloride, confirming the release of the chloride ligand prior to the interaction with proteins (vide infra).

On average, the **Ru-pCy1, 2, 4, and 5** complexes showed that the relative amount of the adducts obtained with SOD is higher than that obtained with RNase, suggesting a significantly higher reactivity with the former target. The spectrum of nonmetalated SOD is included in Figure 3.

Regarding the interaction with SOD (Figure 3), in the case of **Ru-pCy1**, the peak at 15,715 Da corresponds to the native SOD protein; the other two signals at 16,281 and 16,846 Da are adducts bearing one or two [(*p*-cymene)­Ru­(II)**L1**]^2+^ fragments, originated from the detachment of the chloride ligand from metal scaffolds. Similar patterns are also observed for **Ru-pCy2, 4, and 5**, with the respective [(*p*-cymene)­Ru­(II)**L2**]^2+^, [(*p*-cymene)­Ru­(II)**L4**]^2+^, and [(*p*-cymene)­Ru­(II)**L5**]^2+^ metal fragments in the adducts formed with the protein. The only difference in these cases is the stoichiometry of the formed adducts, which does not exceed a 1:1 ratio, whereas for **Ru-pCy1**, it can reach a 2:1 metal/protein binding ratio. A similar behavior was observed between RNase A and each of the four RACs, as evidenced by the recorded spectra shown in Figure S31.

Based on these experiments and physicochemical characterization data, the high reactivity of **Ru-pCy1** is likely due to a combination of factors. First, it readily undergoes chloride ligand replacement, which is essential for promoting protein interactions. Second, its chemical architecture contains an extended aromatic dppn unit, which facilitates interactions with key biological targets, including proteins.

In other words, the reactivity of **Ru-pCy1**, and more in general, that of the other complexes in the series, appears to be finely tuned by a subtle balance between the lability of the chloride ligand and the stereoelectronic characteristics imparted by their chelates.

Consistent with the ESI-MS data, which indicated that the active metal species was the chloride-loss complex, the UV–vis spectra of four of the five compounds exhibited an identical change upon chloride ligand detachment in aqueous solution (Figure S32) over the 24 h period. Notably, **Ru-pCy3** deviated from this behavior. This compound was the sole exception that did not interact with the SOD protein, a phenomenon attributed to its uniquely strong binding affinity for the chloride ligand.

### Antibacterial Evaluation of RAC Complexes

The antibacterial properties of **Ru-pCy1–5** were investigated against *Burkholderia cenocepacia* strains J2315 and K56-2, two well-characterized clinical isolates of this Gram-negative pathogen, as well as against *Bacillus subtilis* 168, which was used as a model for a Gram-positive bacterial strain.

The obtained results are presented as normalized OD_600_ values, reporting the ratio of bacterial growth relative to that of the control (untreated) cells. These are plotted in Figure 4 as a function of the compound concentrations (μg/mL), providing a quantitative assessment of the dose-dependent effects on bacterial growth of the tested ruthenium complexes.

**Figure 4:**
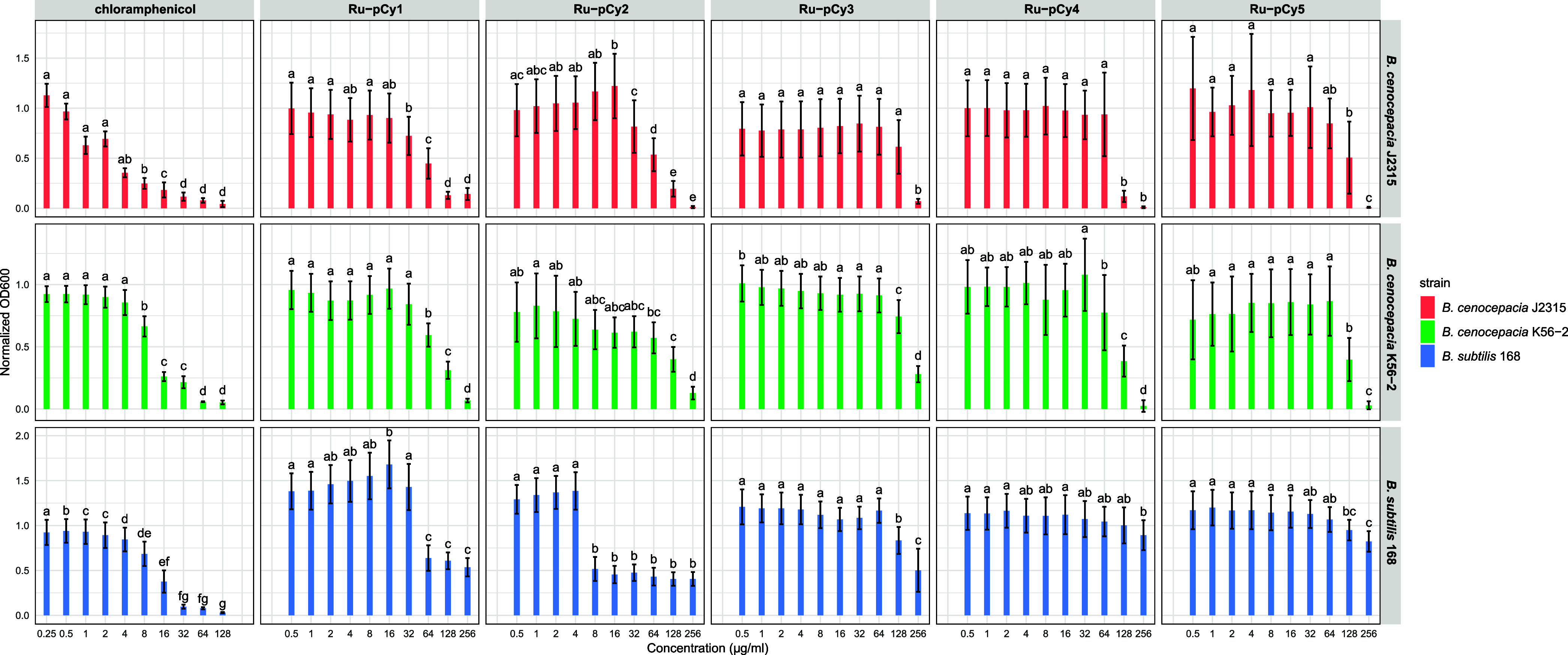
Antibacterial effect of ruthenium compounds **Ru-pCy1–5** evaluated on *B. cenocepacia* J2315, *B. cenocepacia* K56–2, and *B. subtilis* 168 across different drug concentrations. Normalized OD_600_ values are plotted as a function of drug concentrations (μg/mL) on the *X*-axis. Statistical significance between different concentrations is indicated by letters above the bars, derived from Tukey’s posthoc test. Different letters denote significant differences at *p* <0.05.

The analysis of the antibacterial activity profiles of **RupCy1–5** against *B. subtilis* reveals a notable inhibitory effect exerted by **RupCy1** and **RupCy2**. Specifically, these two complexes induced approximately 50% inhibition of bacterial growth at concentrations of 64 and 8 μg/mL, respectively, following a clear dose-dependent trend. This trend was strongly supported by statistical analysis: one-way ANOVA showed extremely high *F*-values for **Ru-pCy2** (*F* = 224.3) and **Ru-pCy1** (*F* = 80.4), both associated with significant *p*-values (*p* <0.0001), confirming a robust effect of concentration on bacterial growth. Although a direct correlation between lipophilicity, hydrolysis of the Ru­(II)–Cl bond, protein-binding ability, and antibacterial efficacy cannot be firmly established based on these data, the enhanced activity of these complexes appears to be particularly favored by their notable protein-binding capacity and lipophilic character. This is evidenced by ESI-MS and Log *P* measurements, which show that these complexes have the highest values of these properties of all metal complexes. Being the different bidentate chelate is the only difference among the tested compounds, it is reasonable to envisage that **L1** and **L2** ligands confer enhanced lipophilicity (Table 1) and protein targeting to **Ru-pCy1** and **Ru-pCy2**, thereby improving the biological efficacy of these ruthenium complexes.

In contrast, inferior activity was found for the more hydrophilic **Ru-pCy4** and **Ru-pCy5** (*F* = 2.8 and *F* = 8.5, respectively, against *B. subtilis*), exhibiting a lower magnitude of response, likely ascribable to their higher hydrophilic character. Lastly, **Ru-pCy3** displayed intermediate antibacterial characteristics with statistical significance (*F* = 35.8), positioning its activity between the two extremes of the series.

A similar pattern was observed against *B. cenocepacia* strains J2315 and K56–2. **Ru-pCy1** and **Ru-pCy2** retained the highest antibacterial efficacy among all tested complexes, with **Ru-pCy1** being the lead compound, inducing approximately 50% inhibition of bacterial growth at a concentration of 32 μg/mL in *B. cenocepacia* J2315. Against J2315, both compounds showed strong concentration effects (*F* = 74.2 for **Ru-pCy1**; *F* = 78.7 for **Ru-pCy2**), while against K56–2, **Ru-pCy1** achieved the highest F-value in the data set (*F* = 129.1), confirming its potency across all tested strains. Although, the observed activities are comparatively lower than those observed against *B. subtilis*, as generally expected due to differences in membrane composition and compound permeability among Gram-positive and Gram-negative bacteria.^54^

Overall, these findings emphasize that structural differences in RACs, arising from the different properties of their bidentate ligands, play a pivotal role in determining their antibacterial activity. The most active compounds in the series, **Ru-pCy1** and **Ru-pCy2**, exhibit the highest efficacy against the Gram-positive and Gram-negative strains tested. This highlights the importance of fine-tuning the physicochemical properties of metal complexes when designing novel and effective antibacterial agents.

## Conclusions

Five RAC complexes of the general formula [(η^6^-arene)­Ru­(L)­X]­PF_6_ (i.e., **Ru-pCy1–5**) have been synthesized and fully characterized. The series included the two newly prepared compounds **Ru-pCy4** and **Ru-pCy5**, together with **Ru-pCy1–3**, to create a small library of complexes with diverse chemical architectures. In particular, bidentate L ligands differed in terms of π-aromatic extension and the presence of hydrophilic morpholine moieties appended to bipyridine through either amide or methylene linkers. The aim was to explore how these structural variations influence the chemical–physical properties of RACs and their potential pharmaceutical relevance.

Following the synthesis, accomplished by small modifications of literature methods for **Ru-pCy1–3**, the five ruthenium complexes were fully characterized and their chemical–physical properties were investigated. Overall, all these compounds exhibit rather favorable physicochemical properties that make them well suited for pharmaceutical use. The UV–vis absorption profiles were in line with those expected for this class of compounds while exhibiting moderate emission. Water–octanol partition coefficients determined at pH 7.4 ranged from −0.66 to +0.91, reflecting the lipophilic or hydrophilic contributions imparted by the different nature of L chelates.

Prior to evaluating their protein-binding activities, we investigated the hydrolysis of the Ru­(II)–Cl bond in RACs, given its recognized role as a preliminary step in binding to target proteins. Spectrophotometric data confirmed that all the compounds readily undergo hydrolysis in water to generate the more reactive aquo species, with the exception of the less reactive **Ru-pCy3**.

The reactivity of RACs toward ribonuclease A (RNase A), superoxide dismutase (SOD), and cytochrome c (CytC), chosen as model proteins to explore the metallodrug–protein interaction at the molecular level, was assessed by ESI-MS. Due to the lack of interaction with CytC, we focused on RNase A and SOD. Except for **Ru-pCy3**, lacking reactivity likely due to a poor combination between lability of the chloride ligand and stereo electronic features imparted by **L3**, **Ru-pCy1–2–4** and **Ru-pCy5** were found to form stable adducts with both proteins, with the highest reactivity exhibited toward SOD. In all cases, the protein adducts contained the characteristic [(*p*-cymene)­Ru­(II)­L]^2+^ fragments (L = **L1**, **L2**, **L4**, **L5**), confirming the key role played by Ru–Cl bond cleavage in the protein interaction. Notably, **Ru-pCy1** exhibited the highest reactivity, forming adducts with SOD with up to two metal fragments; meanwhile, the stoichiometry for the other complexes did not exceed a 1:1 ratio, therefore highlighting the favorable contribution imparted by the dppn ligand to protein binding.

The antimicrobial properties of RACs were then tested against the Gram-positive bacterium *B. subtilis* and the Gram-negative bacterium *B. cenocepacia*. Some significant antibacterial effects were highlighted, which were tentatively correlated with the structural differences among the complexes. Among tested compounds, **Ru-pCy1** and **Ru-pCy2** emerged as the most active agents, exhibiting clear dose-dependent inhibition of bacterial growth in all three strains. Their enhanced biological activity is likely associated with their increased lipophilicity and stronger protein-binding ability, as suggested by Log *P* values and ESI-MS analysis. In contrast, the reduced efficacy of **Ru-pCy4** and **Ru-pCy5** appears to mainly reflect their lower lipophilicity and likely membrane interaction potential.

Although a straightforward and more comprehensive correlation between physicochemical properties, protein interactions, and antibacterial efficacy cannot be given at this stage, these findings underline the key role of rational ligand design in modulating the biological activity of metal-based compounds and confirm that physicochemical tuning can significantly impact antimicrobial performance. Additionally, while ruthenium­(II)–arene complexes are widely known for their anticancer properties, this study contributes to building knowledge of their antibacterial potential. Altogether, it provides a foundation for future developments of ruthenium-based agents with tailored biological properties and encourages further investigation into their mechanisms of action and potential therapeutic applications.

## Experimental Section

### List of Abbreviations

**L1** = dppn = benzo­[*i*]­dipyrido­[3,2-*a*: 2′,3′-*c*]­phenazine, **L2** = DIP = 4,7-diphenyl-1,10-phenanthroline, **L3** = biq = 2,2′-biquinoline, **L4** = 2,2′-bipyridine-4,4′-diylbis­(morpholinomethanone), **L5** = 2,2′-bipyridine -4,4′-diylbis­(morpholinomethylene), PBS = phosphate-buffered saline.

All chemical materials were reagent grade and used without further purification unless otherwise specified. The synthesis of compounds **Ru-pCy2** and **Ru-pCy3** was carried out using 4,7-diphenyl-1,10-phenanthroline (**L2**) and 2,2′-biquinoline (**L3**), purchased from Sigma-Aldrich and utilized directly without additional purification. Benzo­[*i*]­dipyrido­[3,2-*a*: 2′,3′-*c*]­phenazine (**L1**) for the synthesis of complex **Ru-pCy1** was synthesized through Schiff-base condensation between 1,10-phenantroline-5,6-dione and 2,3-diaminonaphthalene, following the procedure described in the literature.^40^ The synthesis of complex **Ru-pCy4** was achieved by preparing the ancillary ligand 2,2′-bipyridine-4,4′-diylbis­(morpholinomethanone) (**L4**), as previously detailed by our research group.^41,55^ In the preparation of compound **Ru-pCy5**, the ligand 2,2′-bipyridine-4,4′-diylbis­(morpholinomethylene) (**L5**) was synthesized for the first time in this work. Briefly, compound **L5** was synthesized using commercially available 2,2′-bipyridine-4,4′-dicarboxylic acid as a starting material. This was first esterified to 4,4′-bis­(carbomethoxy)-2,2′-bipyridine and then reduced to 4,4′-bis­(hydroxymethyl)-2,2′-bipyridine and finally converted into 4,4′-bis­(bromomethyl)-2,2′-bipyridine.^56^ The dibromo derivative was then used in the nucleophilic substitution reaction with morpholine in the presence of potassium carbonate as a base.

#### Synthesis of 2,2′-Bipyridine-4,4′-diylbis­(morpholinomethylene) (**L5**)

A suspension of 4,4′-bis­(bromomethyl)-2,2′-bipyridine (0.24 mmol, 82 mg) in freshly distilled acetonitrile (2.5 mL) was added with morpholine (0.96 mmol, 84 mg) and potassium carbonate (0.62 mmol, 86 mg). The white suspension was stirred at 50 °C overnight, and the progression of the reaction was monitored via thin-layer chromatography on alumina (eluent mixture petroleum ether:ethyl acetate 2:1) until the complete disappearance of the starting material and of the monosubstituted biproduct. Once the reaction had reached completion, the mixture was cooled to room temperature, diluted in dichloromethane (50 mL), and washed with water (10 mL × 3) and once with brine. The crude product was purified by flash chromatography on silica gel using a mixture of dichloromethane and methanol 20:1 to yield compound 2,2′-bipyridine-4,4′-diylbis­(morpholinomethylene) (**L5**) as a white powder in 98% yield.

^1^H NMR (400 MHz, CD_3_CN): δ 8.60 (d, *J*_a‑b_ = 4.8 Hz, 2H, Ha), 8.40 (s, 2H, Hc), 8.36 (d, *J*_b‑a_ = 4.4 Hz, 2H, Hb), 3.67–3.63 (m, 8H, N–CH_2_–CH_2_–O morph), 3.60 (s, 4H, methylene bridge), 2.47–2.42 (m, 8H, N–CH_2_–CH_2_–O morph) ppm.

^13^C NMR (400 MHz, CD_3_CN): δ 156.6, 149.8 Ca, 149.1, 124.8 Cb, 121.4 Cc, 67.2 CH_2_-morph, 62.4 CH_2_-methylene bridge, 54.1 CH_2_-morph ppm.

#### General Procedure for the Synthesis of Compounds **Ru-pCy1–5**

In a general procedure, the commercial precursor dichloro­(*p*-cymene)­ruthenium­(II) dimer was dissolved in a 50/50 v/v mixture of chloroform and methanol at a molar concentration of 0.025 M. Two molar equivalents of the selected polypyridyl ligand were added to the reaction mixture, and the solution was heated at 40 °C, stirred for a period included between 2 and 4 h, depending on the polypyridyl ligand, and monitored by thin layer chromatography on silica. Upon completion, the reaction mixture was cooled to room temperature, and the volume was reduced by half to remove the chloroform. The crude product was precipitated as hexafluorophosphate salts and purified by flash chromatography on silica gel. Different precipitation and purification procedures were adopted depending on the solubility features of the products.

#### Ru-pCy1

The complex was precipitated by the addition of a methanolic solution of 0.01 M potassium hexafluorophosphate and collected by filtration under reduced pressure, washing with methanol and diethyl ether. The crude product was purified by flash chromatography on silica gel using a gradient mixture of dichloromethane/methanol from 20:1 to 15:1. **Ru-pCy1** was obtained as a yellow-orange powder with a yield of 76%.

^1^H NMR (400 MHz, (CD_3_)_2_CO): δ 10.04 (d, *J*_a‑b_ = 4.8 Hz, 2H, Ha), 9.47 (d; *J*_c‑b_ = 8.0 Hz, 2H, Hc), 8.66 (s, 2H, Hd), 8.23 (dd, *J*_b‑c_ = 8.0 Hz, *J*_b‑a_ = 5.2 Hz, 2H, Hb), 8.14–8.09 (m, 2H, He or Hf), 7.57–7.52 (m, 2H, He or Hf), 6.45 (d, *J*_2–3/3–2_ = 6.0 Hz, 2H, H2 or H3), 6.23 (d, J_2–3/3–2_ = 6.4 Hz, 2H, H2 or H3), 2.95 (quint, *J* = 6.8 Hz, 1H, –CH isopropyl), 2.41 (s, 3H, –CH_3_), 1.90 (d, *J* = 6.8 Hz, 6H, –CH_3_ isopropyl) ppm.

^13^C NMR (400 MHz, (CD_3_)_2_CO): δ 157.8 Ca, 149.5, 140.2, 138.4, 136.0 Cc, 135.4, 130.9, 129.1, 128.5, 128.3, 106.7, 103.8, 86.7 C2 or C3, 85.4 C2 or C3, 31.7 –CH isopropyl, 22.1-CH_3_ isopropyl, 18.6 –CH_3_ ppm.

HR-MS (ESI+) *m*/*z*: calcd for C_32_H_26_ClN_4_Ru [M–PF_6_]^+^ 603.08840; measured: 603.08691.

#### Ru-pCy2

For complex **Ru-pCy2**, after the removal of the chloroform from the reaction mixture, precipitation was achieved by addition of an aqueous solution of 0.1 M hexafluorophosphate. The crude product was collected by filtration under reduced pressure, washing with water and diethyl ether. Purification by flash chromatography on silica gel using a mixture of dichloromethane and methanol 20:1 allows the obtainment of complex **Ru-pCy2** as a yellow powder with a yield of 95%.

^1^H NMR (400 MHz, (CD_3_)_2_CO): δ 10.07 (d, *J*_a‑b_ = 5.6 Hz, 2H, Ha), 8.23 (s, 2H, Hc), 8.15 (d, *J*_b‑a_ = 5.6 Hz, Hb), 7.80–7.66 (m, 10H, -phenyl), 6.43 (d, *J*_2–3/3–2_ = 6.4 Hz, 2H, H2 or H3), 6.21 (d, *J*_2–3/3–2_ = 6.4 Hz, 2H, H2 or H3), 2.92 (quint, *J* = 6.8, 1H, –CH isopropyl), 2.35 (s, 3H, –CH_3_), 1.16 (d, J = 7.2 Hz, 6H, –CH_3_ isopropyl) ppm.

^13^C NMR (400 MHz, (CD_3_)_2_CO): δ 156.0 Ca, 151.5, 147.2, 136.0, 130.54 C phenyl, 130.52 C phenyl, 129.8, 129.0, 127.0 Cb, 126.2 Cc, 106.4, 103.3, 86.7 C2 or C3, 85.5 C2 or C3, 31.6 –CH isopropyl, 22.0 –CH_3_ isopropyl, 18.5 –CH_3_ ppm.

HR-MS (ESI+) *m*/*z*: calcd for C_34_H_30_ClN_2_Ru [M–PF_6_]^+^ 603.11355; measured: 603.11794.

#### Ru-pCy3

Precipitation and purification of complex **Ru-pCy3** was obtained by following the same experimental procedure used for complex **Ru-pCy1**. After the isolation of the complex from the reaction mixture, purification was achieved through flash chromatography on silica gel using as an eluent a mixture of dichloromethane and methanol 40:1. **Ru-pCy3** was obtained as an orange powder in a yield of 67%.

^1^H NMR (400 MHz, (CD_3_)_2_CO): δ 9.17 (d, *J*_a‑b_ = 8.8 Hz, 2H, Ha), 8.97 (d, *J*_e‑f/f‑e_ = 8.8 Hz, 2H, He or Hf), 8.90 (d, *J*_e‑f/f‑e_ = 8.8 Hz, 2H, He or Hf), 8.30 (d, *J*_d‑c_ = 8.0 Hz, 2H, Hd), 8.17 (dd, *J* ^1^ = 8.4 Hz, J^2^ = 1.2 Hz, 2H, Hb), 8.00 (dd, *J* = 7.2 Hz, *J* ^2^ = 1.2 Hz, 2H, Hc), 6.05 (d, *J*_2–3/3–2_ = 6.4 Hz, 2H, H2 or H3), 5.94 (d, *J*_2–3/3–2_ = 6.4 Hz, 2H, H2 or H3), 2.46 (s, 3H, –CH_3_), 2.23 (quint, 1H, –CH isopropyl), 0.93 (d, *J* = 7.2 Hz, 6H, –CH_3_ isopropyl) ppm.

^13^C NMR (400 MHz, (CD_3_)_2_CO): δ 157.4, 150.7, 141.9 Ce/Cf, 133.4 Cb, 130.6 Cc, 130.3, 130.2, 129.7 Ca or Cd, 120.8, 106.8, 104.3, 87.3 C2 or C3, 86.8, 31.2 –CH isopropyl, 21.8 –CH_3_ isopropyl, 18.0 –CH_3_ ppm.

HR-MS (ESI+) *m*/*z*: calcd for C_28_H_26_ClN_2_Ru [M–PF_6_]^+^ 527.08225; measured: 527.08192.

#### Ru-pCy4

Precipitation and purification of complex **Ru-pCy4** was obtained by adopting the same experimental procedure used for complex **Ru-pCy2**. The complex **Ru-pCy4** was obtained as a yellow powder with a yield of 77%.

^1^H NMR (400 MHz, (CD_3_)_2_CO): δ 9.70 (d, *J*_a‑b_ = 5.6 Hz, 2H, Ha), 8.70 (s, 2H, Hb), 7.85 (d, *J*_b‑a_ = 7.2 Hz, 2H, Hb), 6.28 (d, *J*_2–3/3–2_ = 6.4 Hz, 2H, H2 or H3), 6.07 (d, *J*_2–3/3–2_ = 6.4 Hz, 2H, H2 or H3), 3.75 (br s, 8H, N–CH_2_–CH_2_–O morph), 3.64 (br s, 4H, N–CH_2_–CH_2_–O morph), 3.97 (br s, 4H, N–CH_2_–CH_2_–O morph), 2.89 (quint, 1H, –CH isopropyl), 2.30 (s, 3H, –CH_3_), 1.16 (d, *J* = 7.2 Hz, 6H, –CH_3_ isopropyl) ppm.

^13^C NMR (400 MHz, (CD_3_)_2_CO): δ 165.6 Ca, 156.7, 155.7, 147.7, 125.8 Cb, 122.6 Cc, 107.0, 103.8, 87.0 C2 or C3, 85.8 C2 or C3, 66.9 C_sec_ morph, 66.7 C_sec_ morph, 48.2 C_sec_ morph, 42.7 C_sec_ morph, 31.6 –CH isopropyl, 22.0 –CH_3_ isopropyl, 18.4 ppm of –CH_3_.

HR-MS: (ESI+) *m*/*z*: calcd for C_30_H_36_ClN_4_O_4_Ru [M–PF_6_]^+^ 653,14,631, measured: 653.14376.

#### Ru-pCy5

In the case of complex **Ru-pCy5**, the addition of a methanolic solution of potassium hexafluorophosphate 0.01 M does not allow the precipitation of the complex; thus, the solution was evaporated to dryness and the crude yellow oily product was dissolved in dichloromethane and washed twice with water and once with brine. After evaporation of the organic phase, flash chromatography on silica gel using dichloromethane and methanol 30:1 allows the obtainment of complex **Ru-pCy5** as a yellow hygroscopic powder with a yield of 70%.

^1^H NMR (400 MHz, (CD_3_)_2_CO): δ 9.52 (d, *J*_a‑b_ = 5.6 Hz, 2H, Ha), 8.58 (s, 2H, Hc), 7.83 (d, *J*_b‑a_ = 5.2 Hz, 2H, Hb), 6.22 (d, *J*_2–3/3–2_ = 6.4 Hz, 2H, H2 or H3), 5.98 (d, *J*_2–3/3–2_ = 6.4 Hz, 2H, H2 or H3), 3.84 (s, 4H, methylene bridge), 3.72–3.66 (m, 8H, N–CH_2_–CH_2_–O morph), 2.78 (quint, 1H, –CH isopropyl), 2.56–2.50 (m, 8H, N–CH_2_–CH_2_–O morph), 2.31 (s, 3H, –CH_3_), 1.10 (d, *J* = 6.8 Hz, –CH_3_ isopropyl) ppm.

^13^C NMR (400 MHz, (CD_3_)_2_CO): δ 155.9 Ca, 155.4, 153.7, 127.8 Cb, 123.8 Cc, 105.5, 104.1, 87.1C2 or C3, 85.0C2 or C3, 67.1 C_sec_ morph, 61.4 –CH isopropyl, 54.2 C_sec_ morph, 36.8, 34.8, 33.2, 32.3, 32.2, 31.5, 25.6, 23.0, 21.9 –CH_3_ isopropyl, 18.5 –CH_3_, 14.0 ppm.

HR-MS: (ESI+) *m*/*z*: calcd. for C_30_H_40_ClN_4_O_2_Ru [M-PF_6_]^+^ 625,18,778, measured: 625.18585.

### NMR Spectroscopy

NMR spectra of compounds were recorded on a Bruker Avance 400 MHz instrument.

### UV–Visible Spectroscopy

Spectrofluorimetric-grade solvents were used for the preparation of all solutions. The PBS solution (pH 7.4, KCl 2.7 mM, NaCl 140 mM, phosphate 10 mM) was prepared according to the standard procedure. RACs were dissolved in CH_3_CN and then diluted to reach a final concentration of 1 × 10^–5^ M in CH_3_CN/buffer solution (CH_3_CN 1% v/v). A PerkinElmer Lambda 900 spectrophotometer was employed to record the UV–Visible absorption spectra using 10 mm path-length quartz cuvettes.

### Determination of Distribution Coefficients (log *P*_7.4_)

The octanol–water distribution coefficients were determined at pH 7.4 using the shake-flask method.^57,58^ Briefly, solutions of the tested compounds (16 μM) in phosphate buffer (pH 7.4) were mixed with *n*-octanol in flasks at three different PBS:*n*-octanol ratios (1:1, 0.5:1, 2:1). The mixtures were manually shaken to allow equilibration between the organic and aqueous phases. Subsequently, phase separation was achieved by centrifugation at 4000 rpm for 10 min. The concentrations of ruthenium compounds in both phases were then determined spectrophotometrically. The log *P*_7.4_ values were calculated as the mean value of the log­[**Ru-pCy1–5**]­oct/[**Ru-pCy1–5**]_PBS/4_ values obtained from the three different PBS:*n*-octanol ratio samples for each complex.

### Aquation of RAC Complexes

Hydrolysis of halido complexes was followed spectroscopically, registering the UV–vis spectra of aqueous solutions at a neutral pH of metal complexes (10 μM) at increasing times, over a total period of 12 h, at 298 K. Plots of the changes at MLCT maximum wavelengths with time were computed fitted using a pseudo-first-order rate model to give the half-lives (*t*_1/2_) and rate constants (*k*_water_) reported in Table 1.

The acid–base behavior of the coordinated water ligand to the metal scaffold was also considered. To this aim, the UV–vis spectra of batchwise aqueous solutions of the lead compound **Ru-pCy1** at different pH at 298 K were registered, and the changes in the MLCT region were monitored. The fitting of the absorbance maxima variation at 435 nm to the Henderson–Hasselbalch equation gave a p*K*_a_ of 8.3 ± 0.1, indicating the predominance at neutral pH of the bipositively charged aqua species of the complex. A slightly higher value (8.82 ± 0.08) was instead determined potentiometrically under mixed solvent conditions (H_2_O/EtOH 4:1 v/v, NaClO_4_ 0.1 M, 298 K, ligand concentration 1 mM),^59^ required to ensure the full solubilization of the complex.

### ESI-MS Experiments

Sample preparation: stock solutions of Bovine Superoxide Dismutase SOD) (Merck), Bovine Pancreatic Ribonuclease A (Merck), and Horse Heart Cytochrome c (Merck) 10^–3^ M were prepared by dissolving the lyophilized protein in LC–MS-grade water. Stock solutions 10^–2^ M RACs were prepared by dissolving the samples in acetonitrile. For the experiments, an aliquot of the stock solutions of the selected protein was mixed with aliquots of each ruthenium compounds at protein-to-metal ratio 1:3 and diluted with LC–MS-grade water to 100 μM final protein concentration. The mixtures were incubated at 37 °C up to 24 h. After the incubation time, the protein solutions were sampled and diluted to a final protein concentration of 500 nM using LC–MS-grade water and adding 0.1% v/v of formic acid just before the infusion in the mass spectrometer.

Instrumental parameters: the ESI mass spectra were acquired through direct infusion at 7 μL min^–1^ flow rate in a TripleTOF 5600+ high-resolution mass spectrometer (Sciex, Framingham, MA, U.S.A.), equipped with a DuoSpray interface operating with an ESI probe.

The ESI source parameters were as follows:

RNase: positive polarity, Ionspray Voltage Floating 5500 V, Temperature 0, Ion source Gas 1 (GS1) 40 L/min; Ion source Gas 2 (GS2) 0; Curtain Gas (CUR) 15 L/min, Declustering Potential (DP) 100 V, Collision Energy (CE) 10 V, acquisition range 1000–2600 *m*/*z*.

SOD: positive polarity, IonSpray Voltage Floating 5500 V, Temperature 0, Ion source Gas 1 (GS1) 40 L/min; Ion source Gas 2 (GS2) 0; Curtain Gas (CUR) 15 L/min, Declustering Potential (DP) 200 V, Collision Energy (CE) 10 V, acquisition range 1500–3500 *m*/*z*.

CytC: positive polarity, IonSpray Voltage Floating −4500 V, Temperature 400, Ion source Gas 1 (GS1) 40 L/min; Ion source Gas 2 (GS2) 30; Curtain Gas (CUR) 25 L/min, Declustering Potential (DP) 100 V, Collision Energy (CE) 10 V, acquisition range 800–3000 *m*/*z*.

For acquisition, Analyst TF software 1.7.1 (Sciex) was used, and deconvoluted spectra were obtained by using the Bio Tool Kit microapplication v.2.2 embedded in Peak-ViewTM software v.2.2 (Sciex).

### Antibacterial Studies

Antimicrobial susceptibility was assessed in the following strains: *B. cenocepacia* K56-2 and J2315 and *B. subtilis* 168. For each bacterial strain, three independent biological replicates were prepared by inoculating Mueller–Hinton Broth (MHB) into sterile culture tubes, followed by overnight incubation at 37 °C with shaking at 130 rpm. Cultures were diluted to a final OD_600_ of 0.05 per well in 96-well microplates (Sarstedt AG & Co.) and exposed to 10 serial 2-fold dilutions of the compounds, ranging from 256 μg/mL to 0.5 μg/mL. Each biological replicate included three technical replicates. Growth inhibition was assessed visually and quantified by measuring OD_600_ after 24 h for *B. subtilis* 168 and after 48 h for *B. cenocepacia* strains K56-2 and J2315 using a Tecan Infinite 200 PRO plate reader. The minimum inhibitory concentration (MIC) values are reported in Table S1.

## Supplementary Material



## Notes

^1^H, ^13^C, ^1^H^1^H–COSY, ^1^H^13^C-HSQC NMR spectra, HR-ESI-MS, UV–vis spectra in PBS, UV–vis spectra in aqueous solution with and without the addition of AgNO_3_ and UV–vis spectra in aqueous solutions with SOD for all compounds; UV–vis spectra in aqueous solution at different pH values of **Ru-pCy**; deconvoluted ESI-Q-TOF spectra of RNase A with **Ru-pCy1**, **2**, **4**, and **5**; and table of MIC values of tested compounds (PDF)
