# Supplementary material for: Playing with Structural Parameters in the Design of Ruthenium(II)–p‑Cymene Complexes as Potential Antibacterial Agents
Source: Inorg Chem. 2025 Nov 19;64(48):23414–25. doi: 10.1021/acs.inorgchem.5c03505 (PMC12690573; doi:10.1021/acs.inorgchem.5c03505)
Supplement: Supplementary file 1 [file ic5c03505_si_001.pdf]

SUPPORTING INFORMATION

Playing with structural parameters in the design of ruthenium(II)–p-cymene complexes as potential antibacterial agents

Gina Elena Giacomazzo[a], Valentina Ceccherini[a], Valentina Vitali[a], Luca Conti[a], Francesca Vaccaro[b], Francesca Coscione[b], Elena Perrin[b], Marco Fondi[b], Lara Massai[a]*, Claudia Giorgi[a‡], Luigi Messori[a‡]*

[a] Department of Chemistry "*Ugo Schiff*", University of Florence, Via della Lastruccia 3, 50019, Sesto Fiorentino (FI), Italy.

[b] Department of Biology, University of Florence, Via Madonna del Piano 6, 50019, Sesto Fiorentino (FI), Italy.

‡ co-last authors

* corresponding authors

Corresponding author e-mail: luca.conti@unifi.it , lara.massai@unifi.it

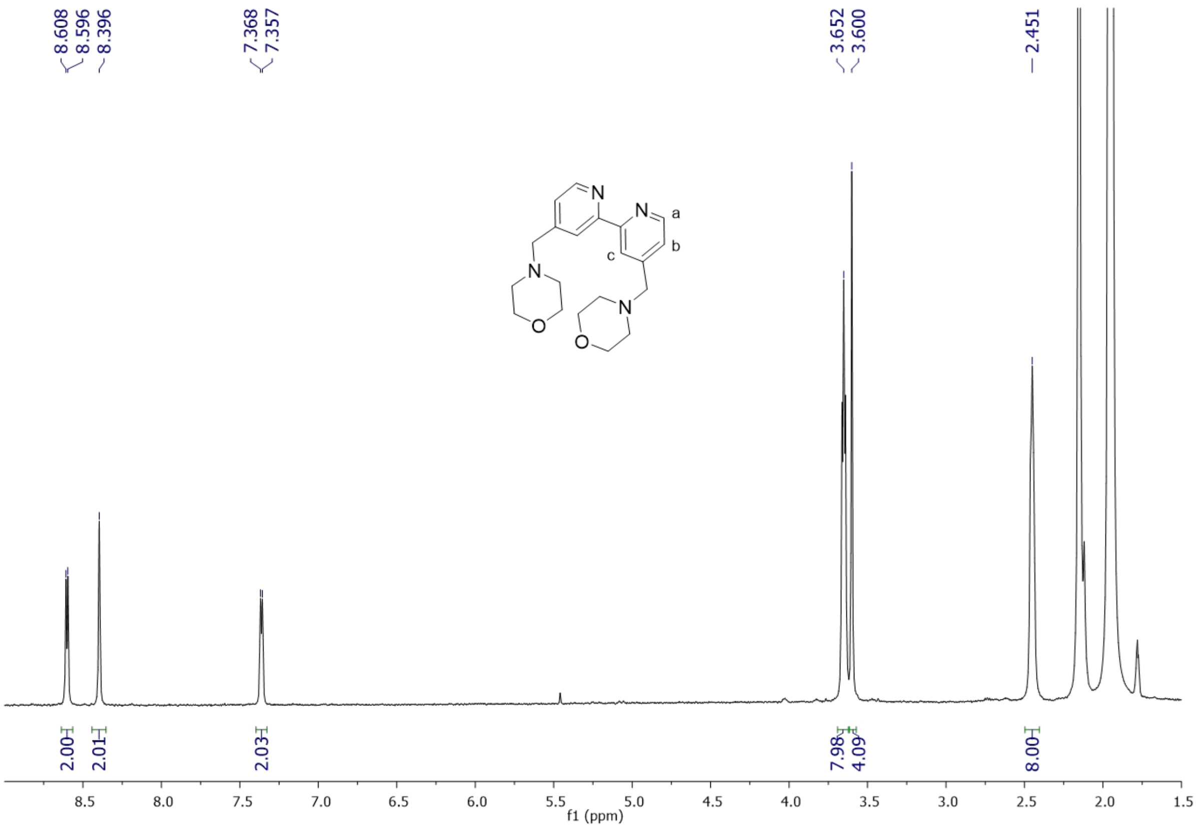

Figure S1. ^{1}H-NMR (400 MHz) spectrum of compound **L5** recorded in CD_3CN.

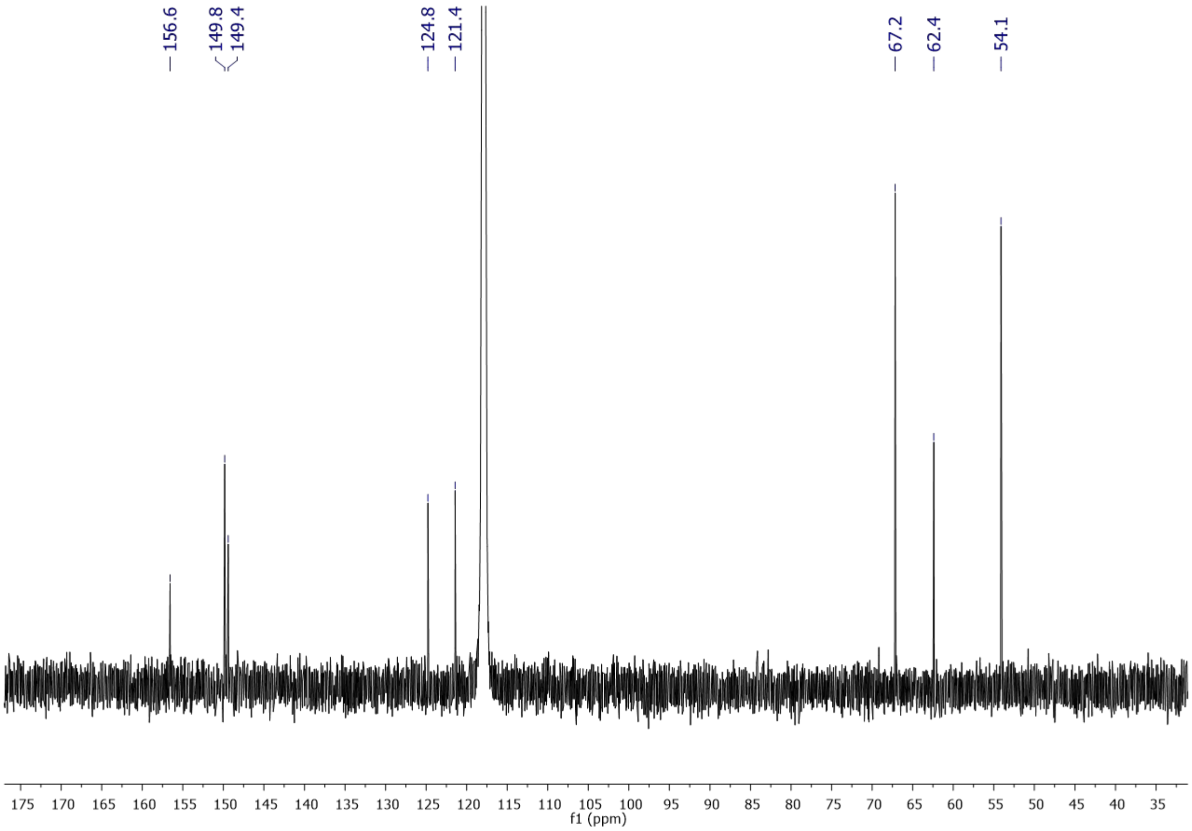

Figure S2. ^{13}C-NMR (100 MHz) spectrum of compound **L5** recorded in CD_3CN.

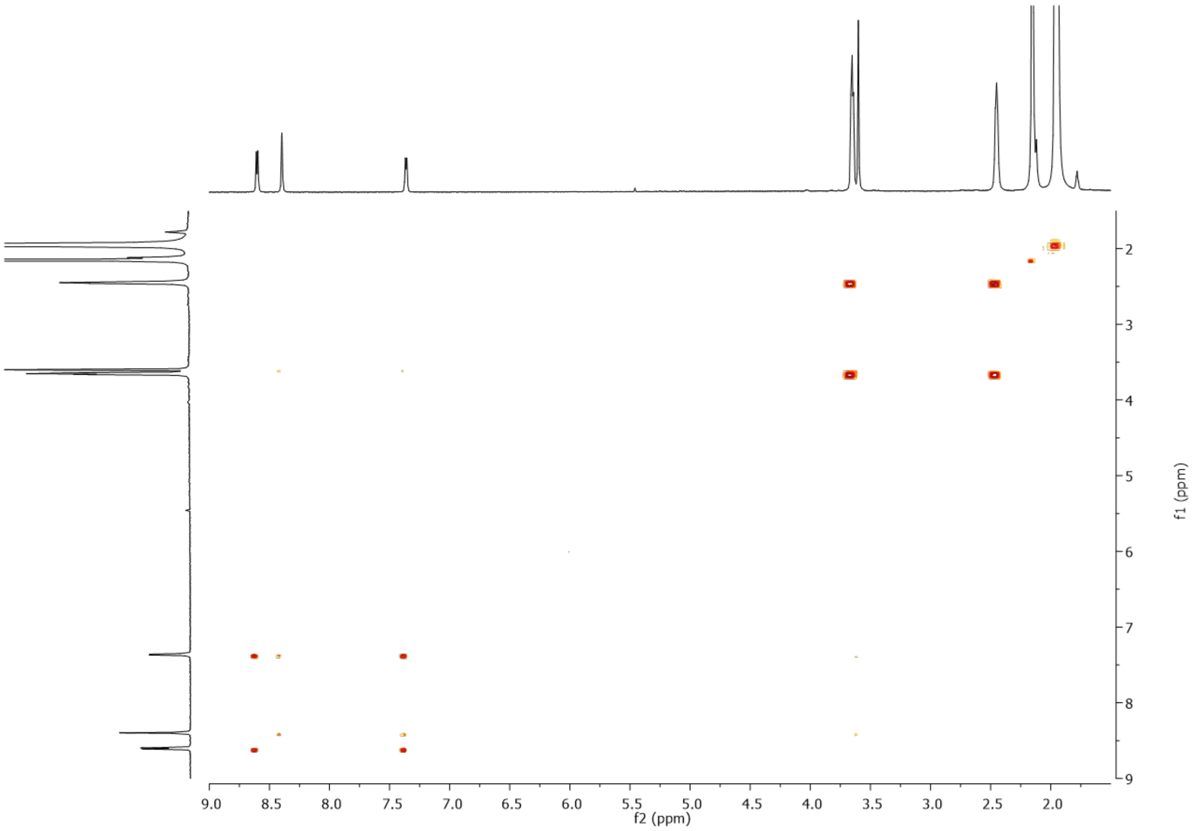

Figure S3. ^{1}H^{1}H-COSY spectrum of compound **L5** recorded in CD_3CN.

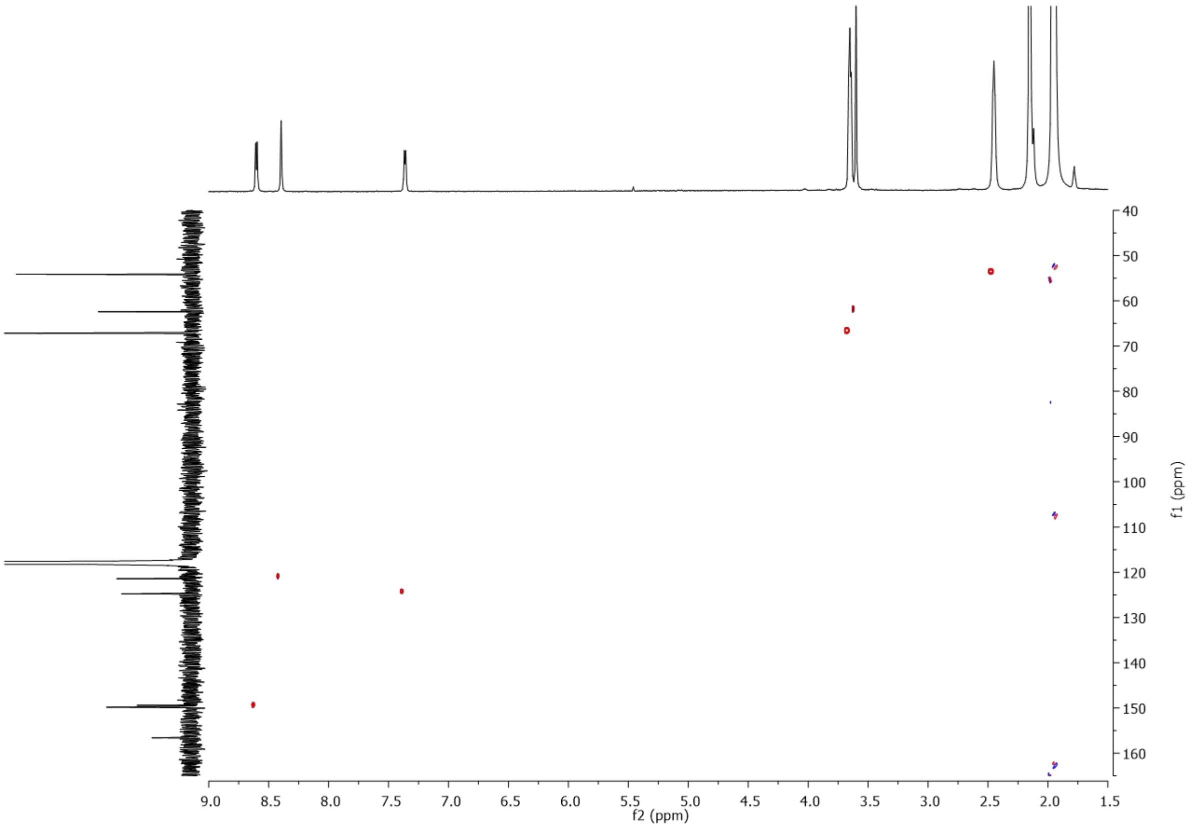

Figure S4. $^{1}H^{13}C$-HSQC spectrum of compound **L5** recorded in CD_3CN.

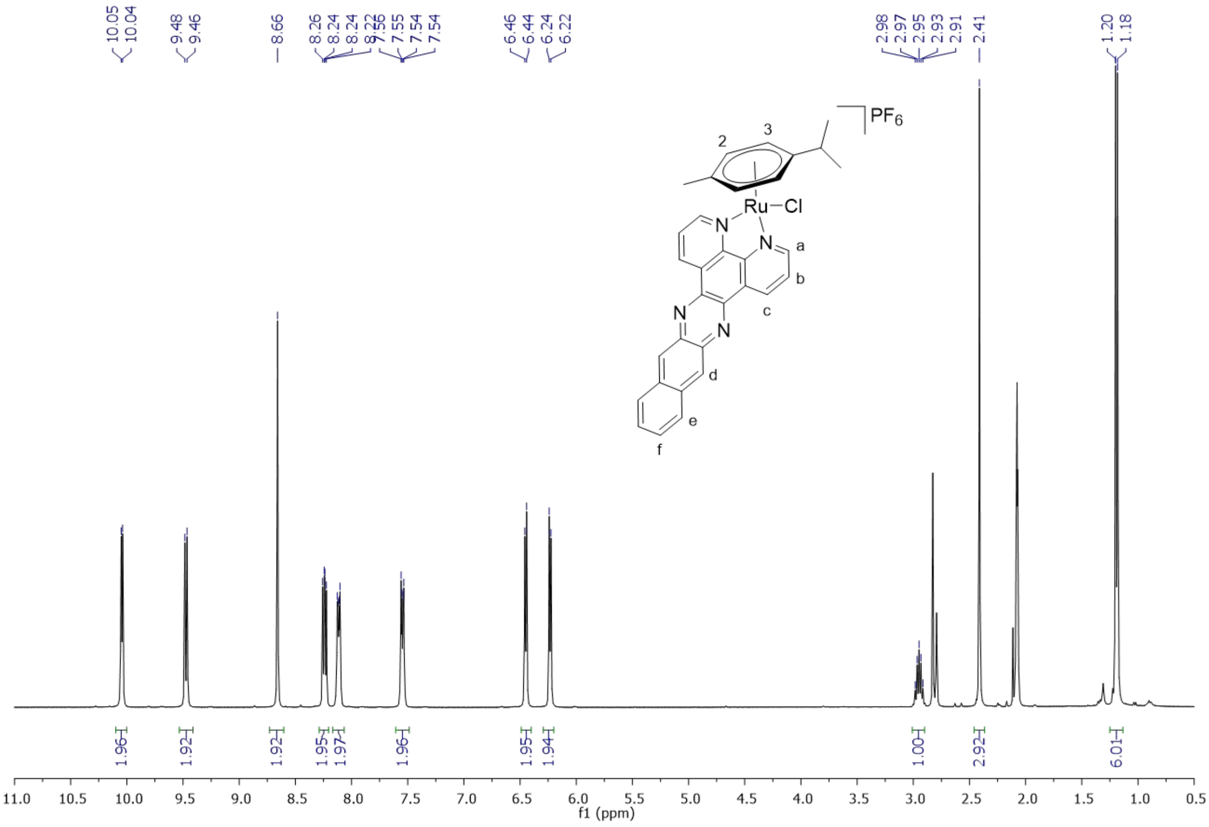

Figure S5. ^{1}H-NMR (400 MHz) spectrum of compound **Ru-pCy1** recorded in $(CD_3)_2CO$.

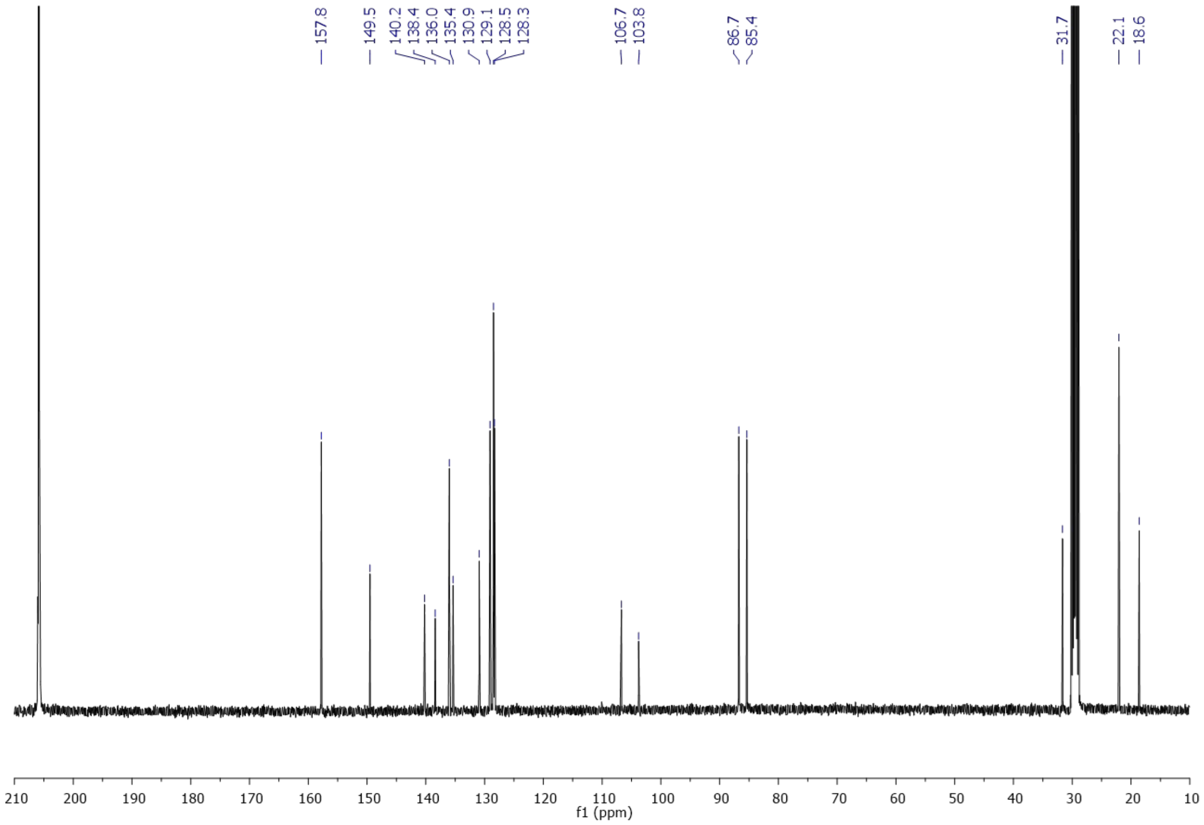

Figure S6. ^{13}C-NMR (100 MHz) spectrum of compound **Ru-pCy1** recorded in $(CD_3)_2CO$.

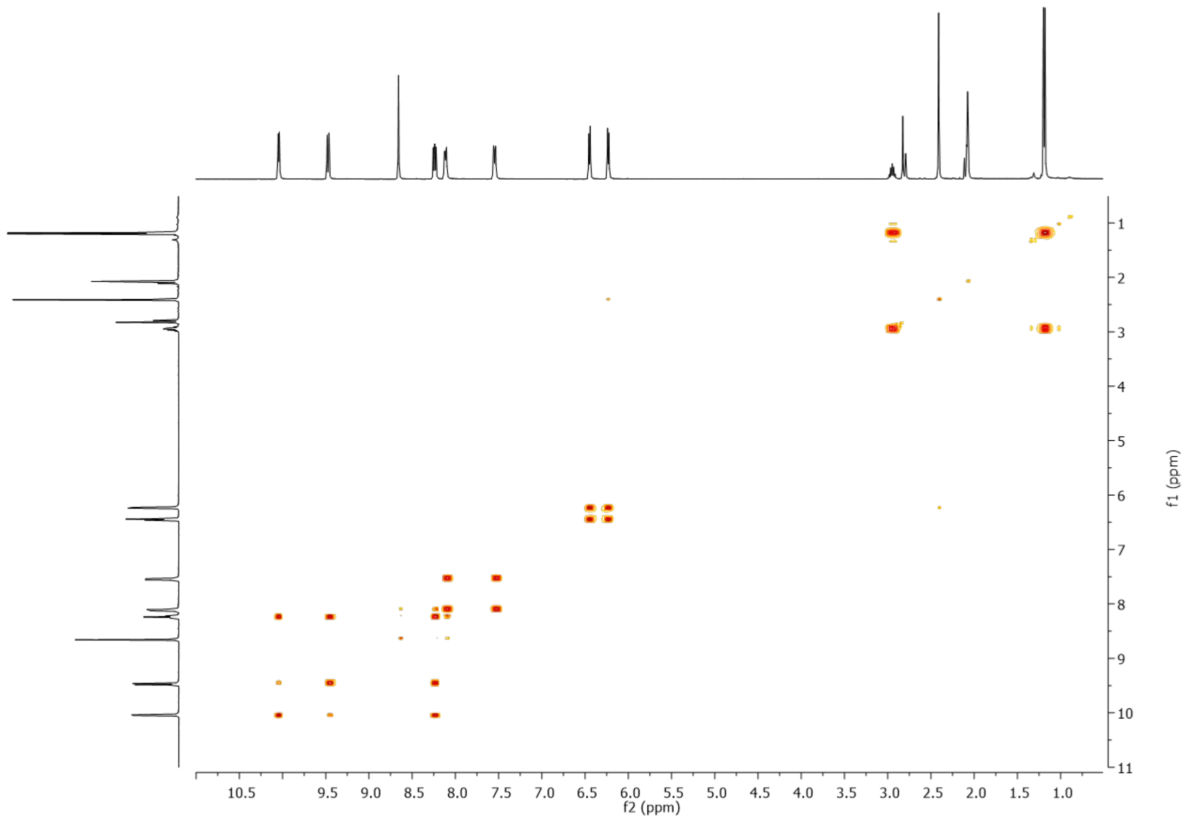

Figure S7. $^{1}H^{1}H$-COSY spectrum of compound **Ru-pCy1** recorded in $(CD_3)_2CO$.

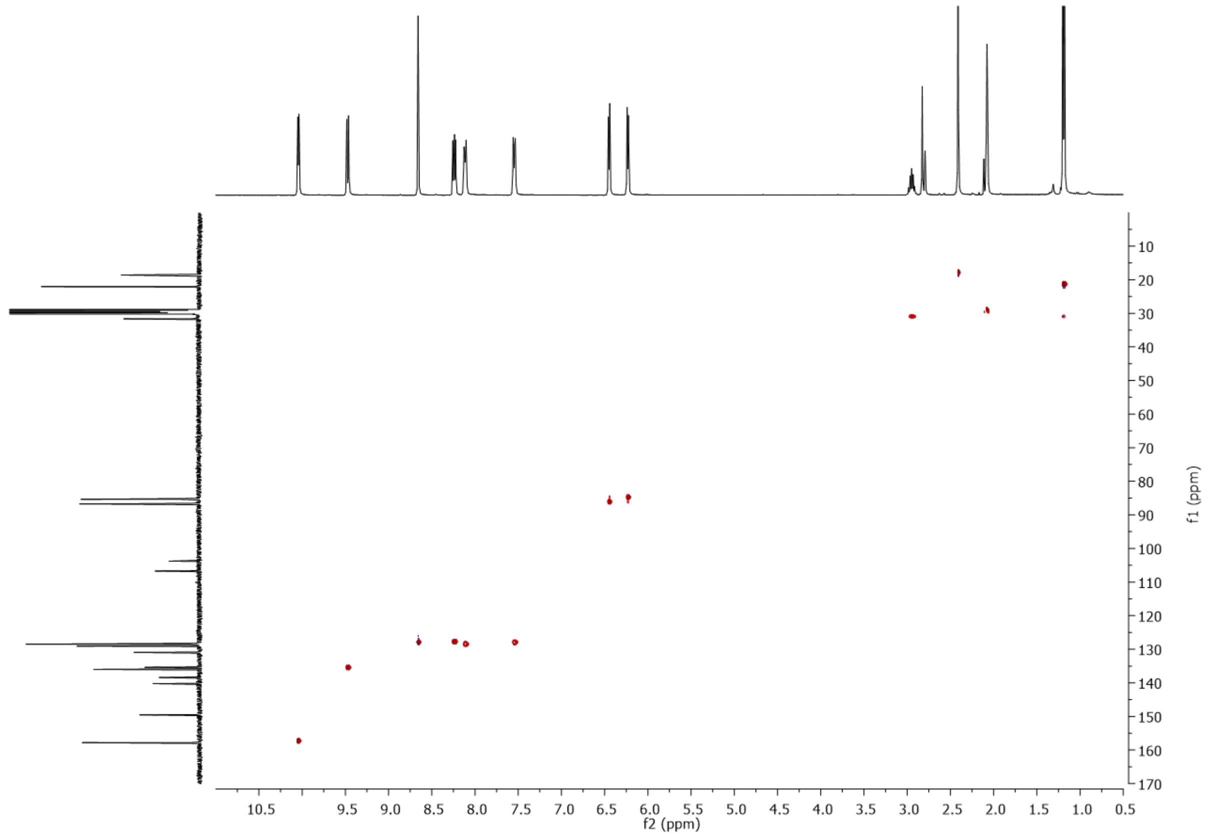

Figure S8. $^{1}H^{13}C$-HSQC spectrum of compound **Ru-pCy1** recorded in $(CD_3)_2CO$.

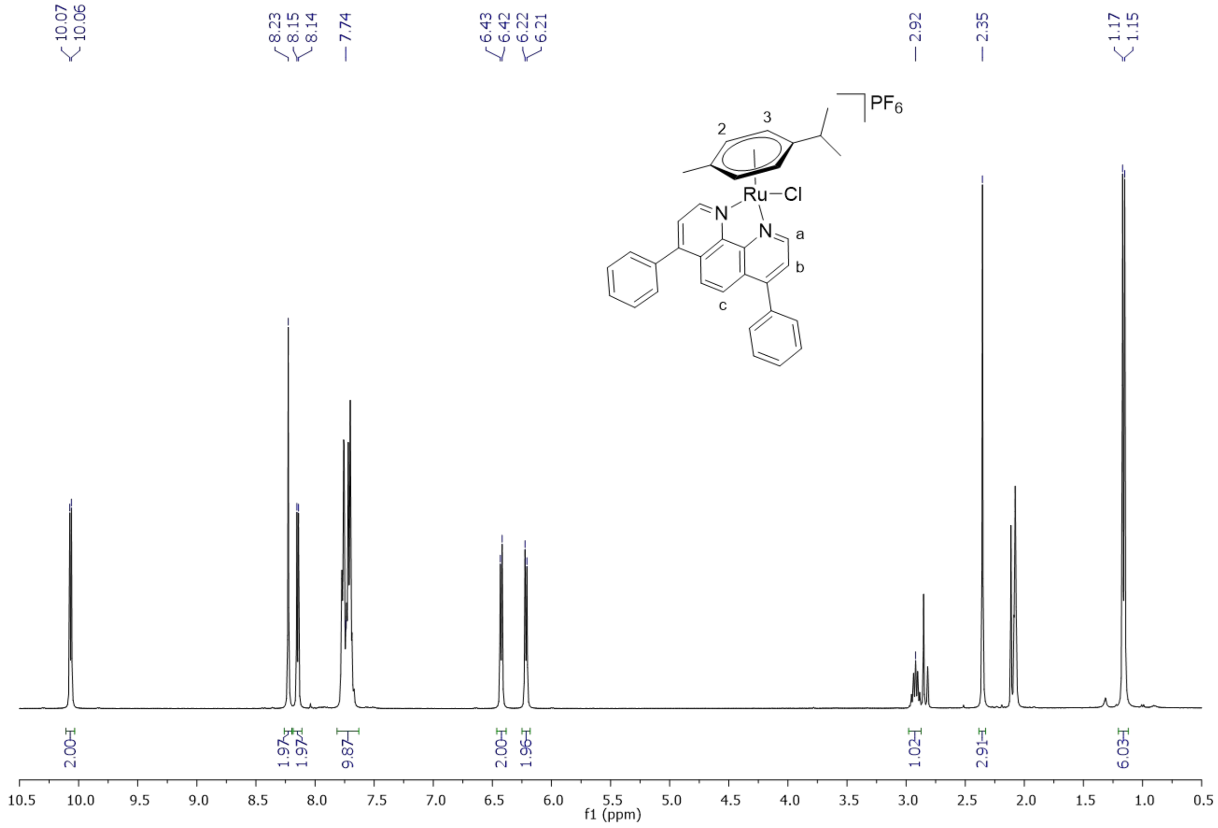

Figure S9. ^{1}H-NMR (400 MHz) spectrum of compound **Ru-pCy2** recorded in $(CD_3)_2CO$.

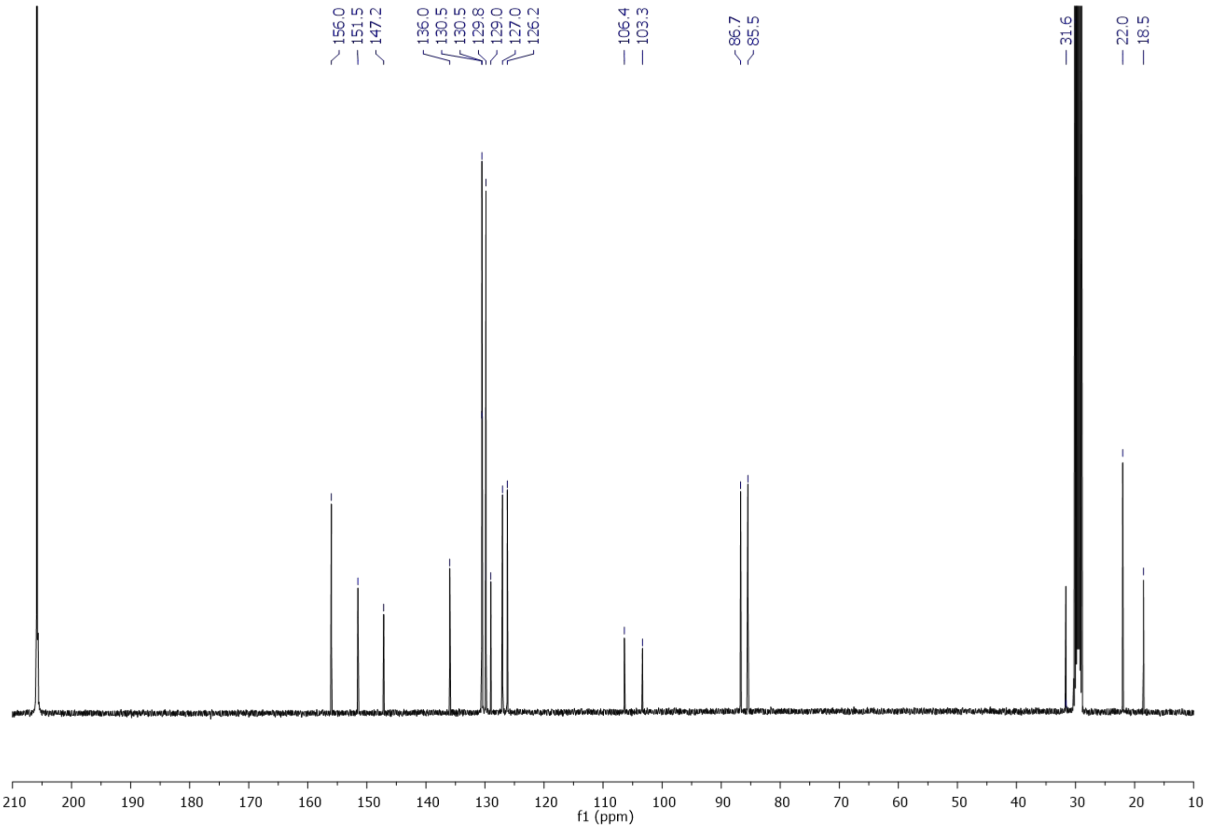

Figure S10. ^{13}C-NMR (100 MHz) spectrum of compound **Ru-pCy2** recorded in $(CD_3)_2CO$.

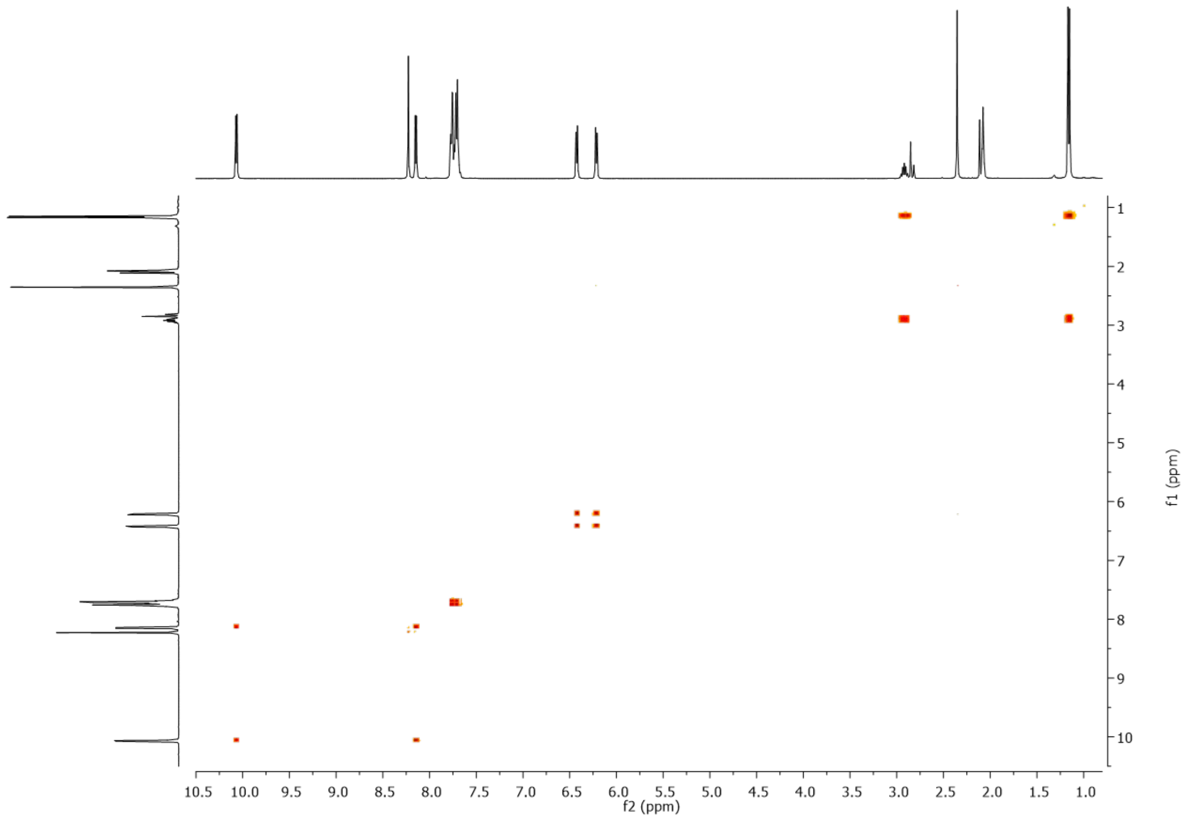

Figure S11. $^{1}H^{1}H$-COSY spectrum of compound **Ru-pCy2** recorded in $(CD_3)_2CO$.

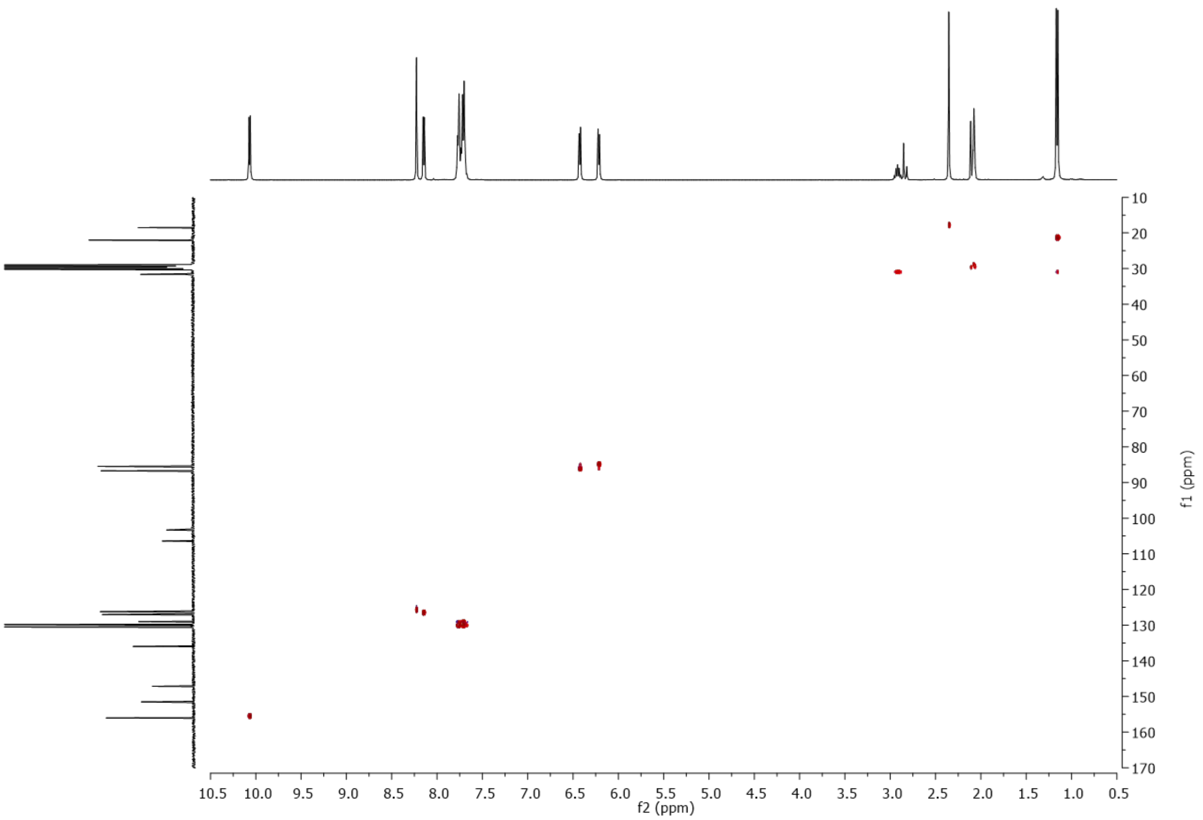

Figure S12. $^{1}H^{13}C$-HSQC spectrum of compound **Ru-pCy2** recorded in $(CD_3)_2CO$.

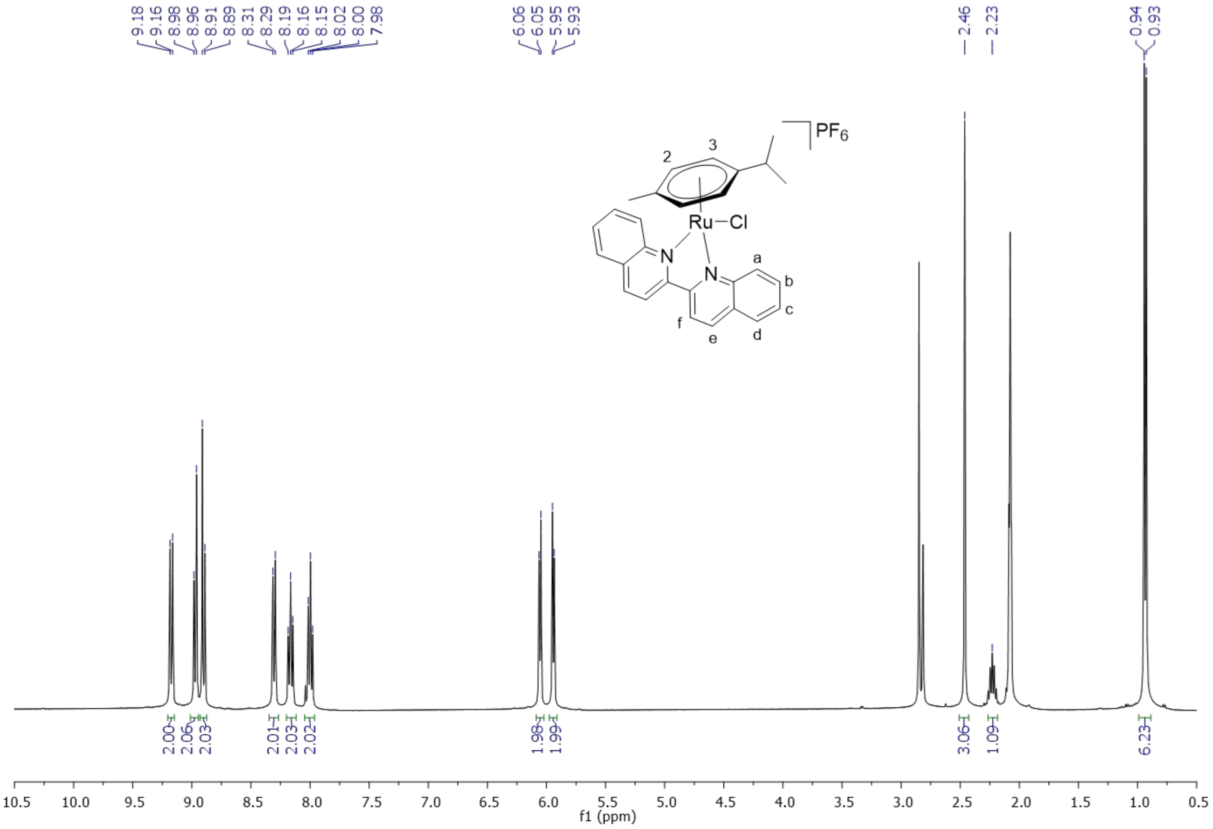

Figure S13. ^{1}H-NMR spectrum of compound **Ru-pCy3** recorded in $(CD_3)_2CO$.

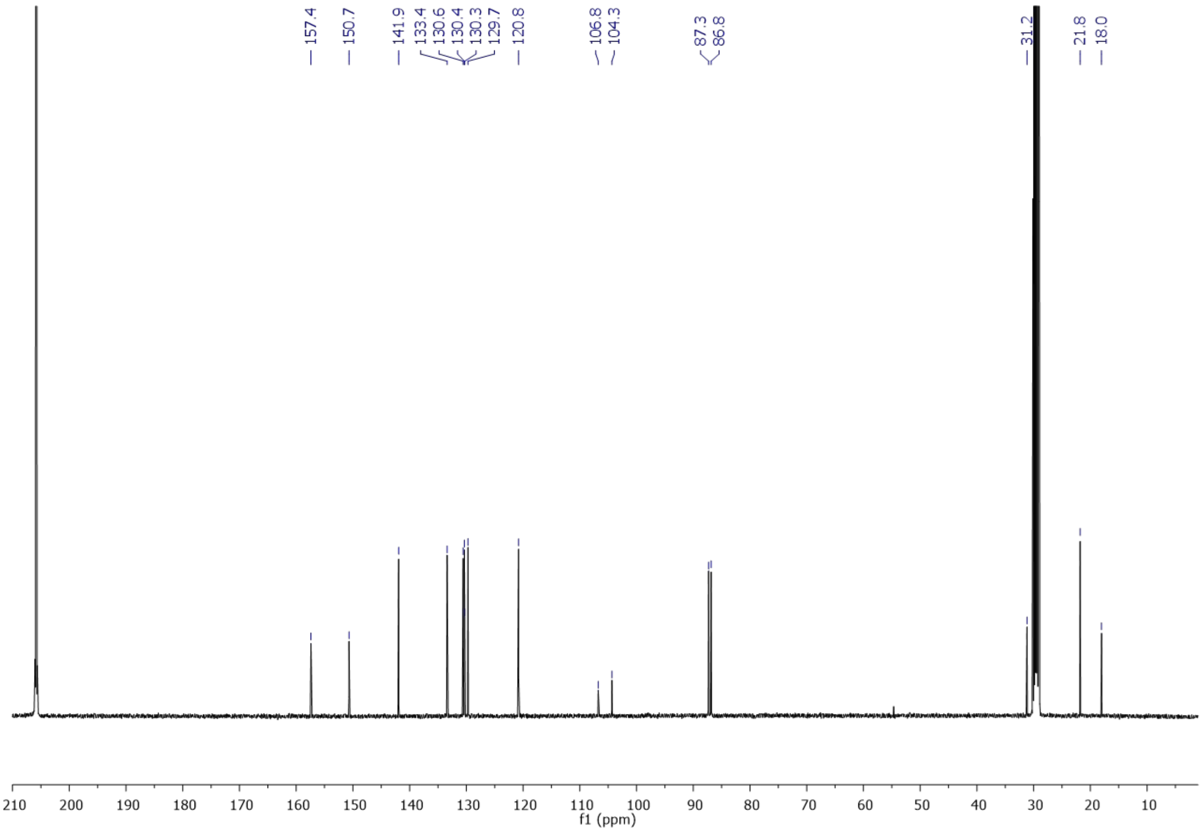

Figure S14. ^{13}C-NMR (100 MHz) spectrum of compound **Ru-pCy3** recorded in $(CD_3)_2CO$.

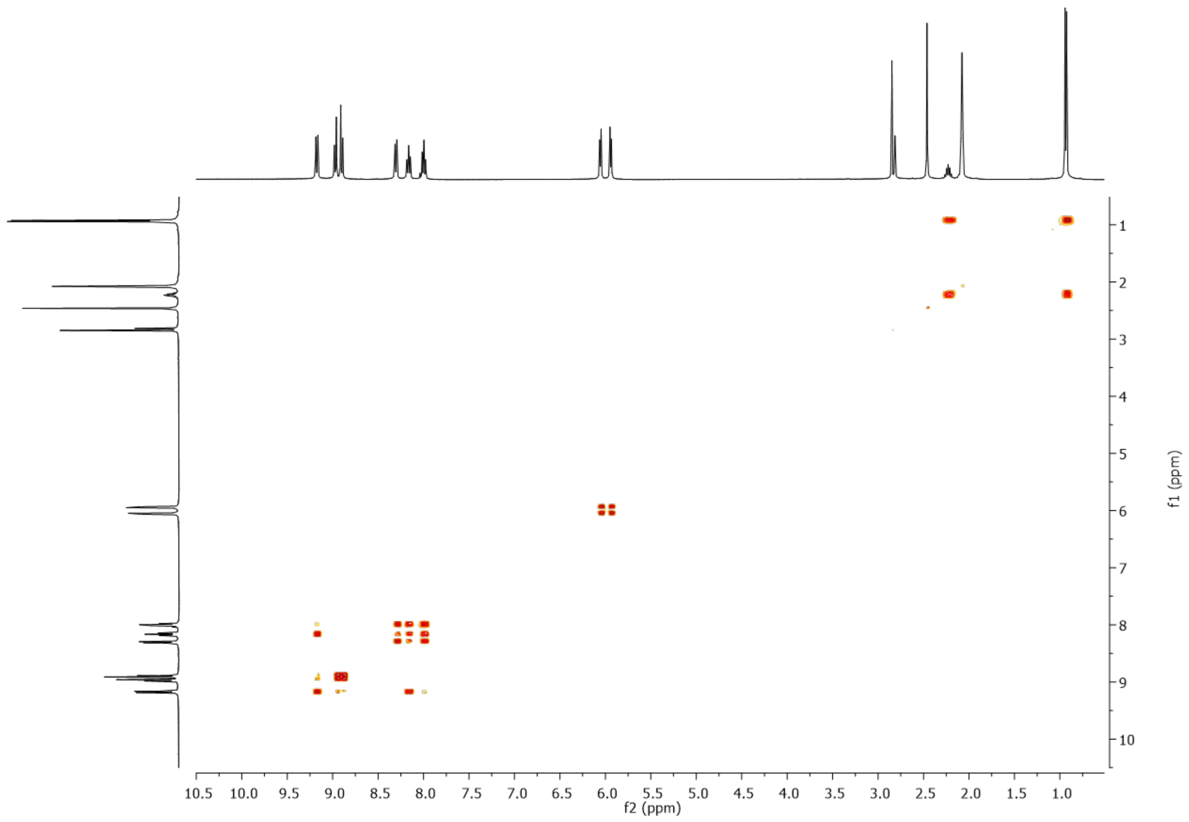

Figure S15. $^1H^1H$-COSY spectrum of compound **Ru-pCy3** recorded in $(CD_3)_2CO$.

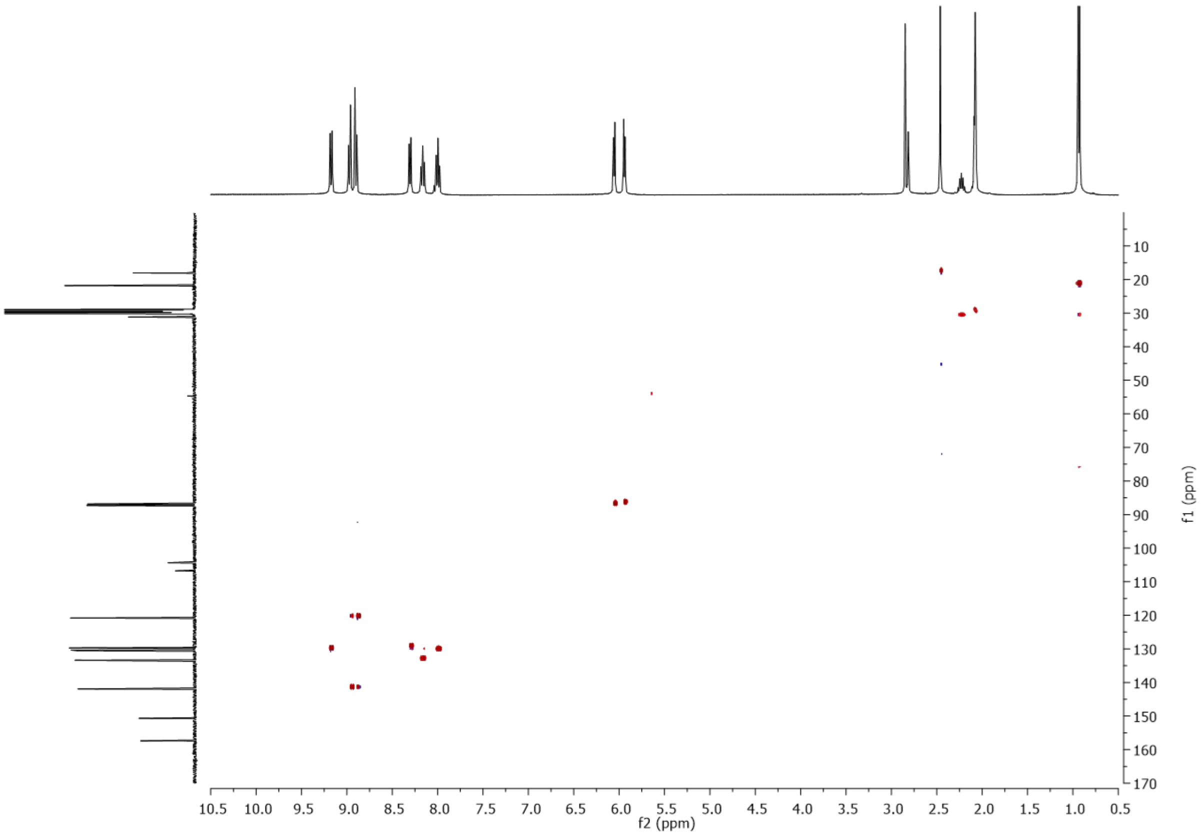

Figure S16. ^{1}H^{1}H-HSQC spectrum of compound **Ru-pCy3** recorded in $(CD_3)_2CO$.

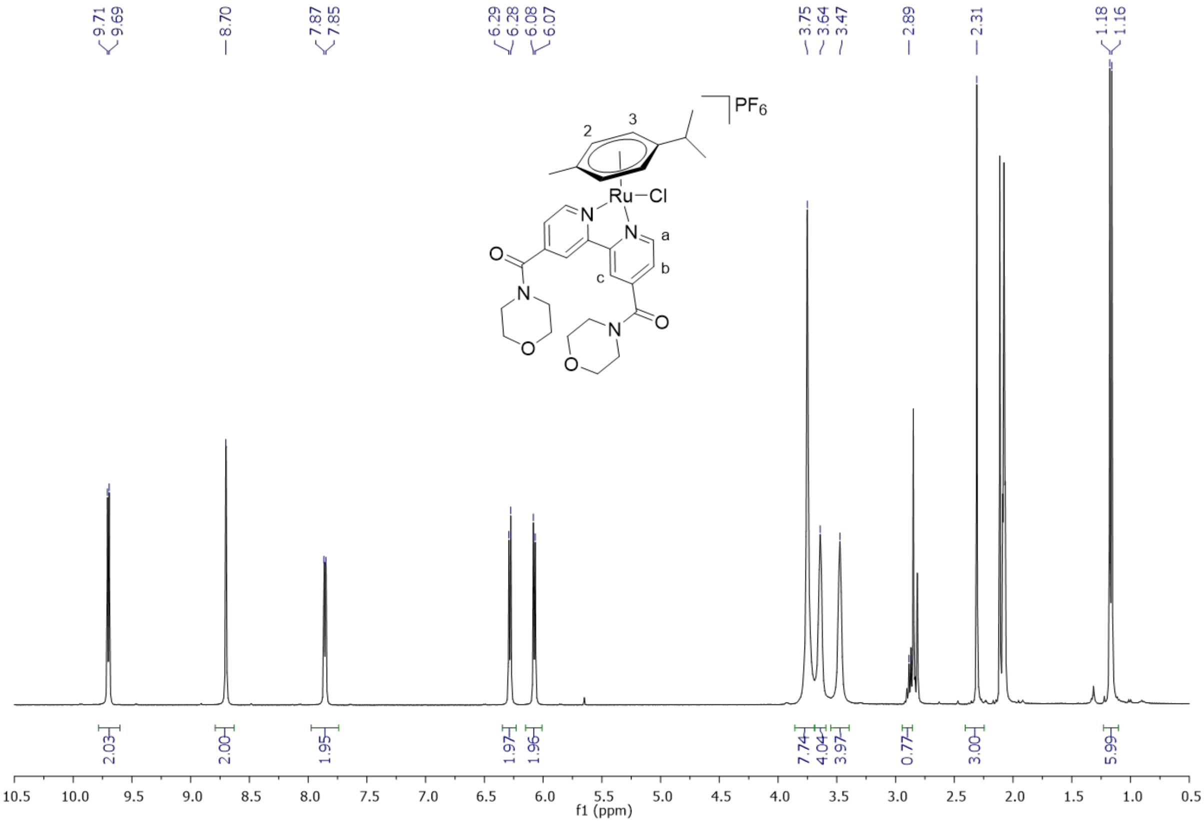

Figure S17. ^{1}H-NMR (400 MHz) spectrum of compound **Ru-pCy4** recorded in $(CD_3)_2CO$.

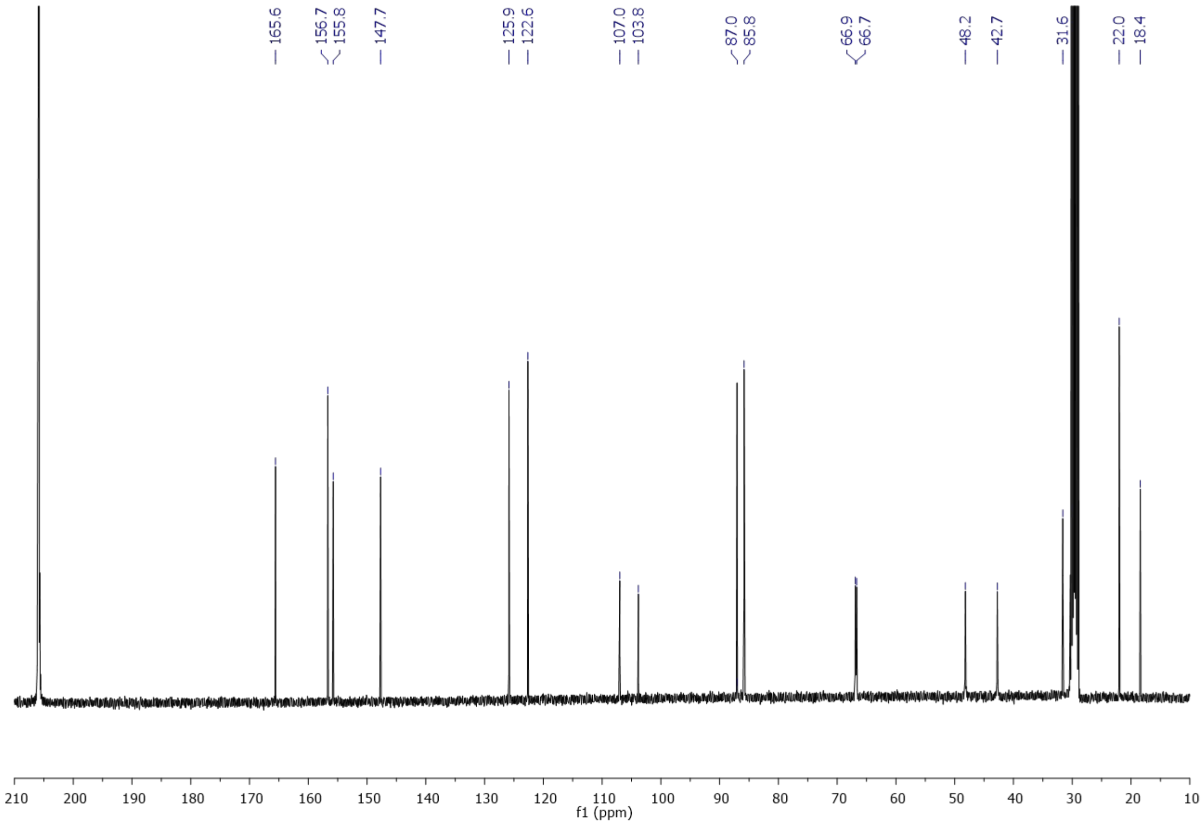

Figure S18. ^{13}C-NMR (100 MHz) spectrum of compound **Ru-pCy4** recorded in $(CD_3)_2CO$.

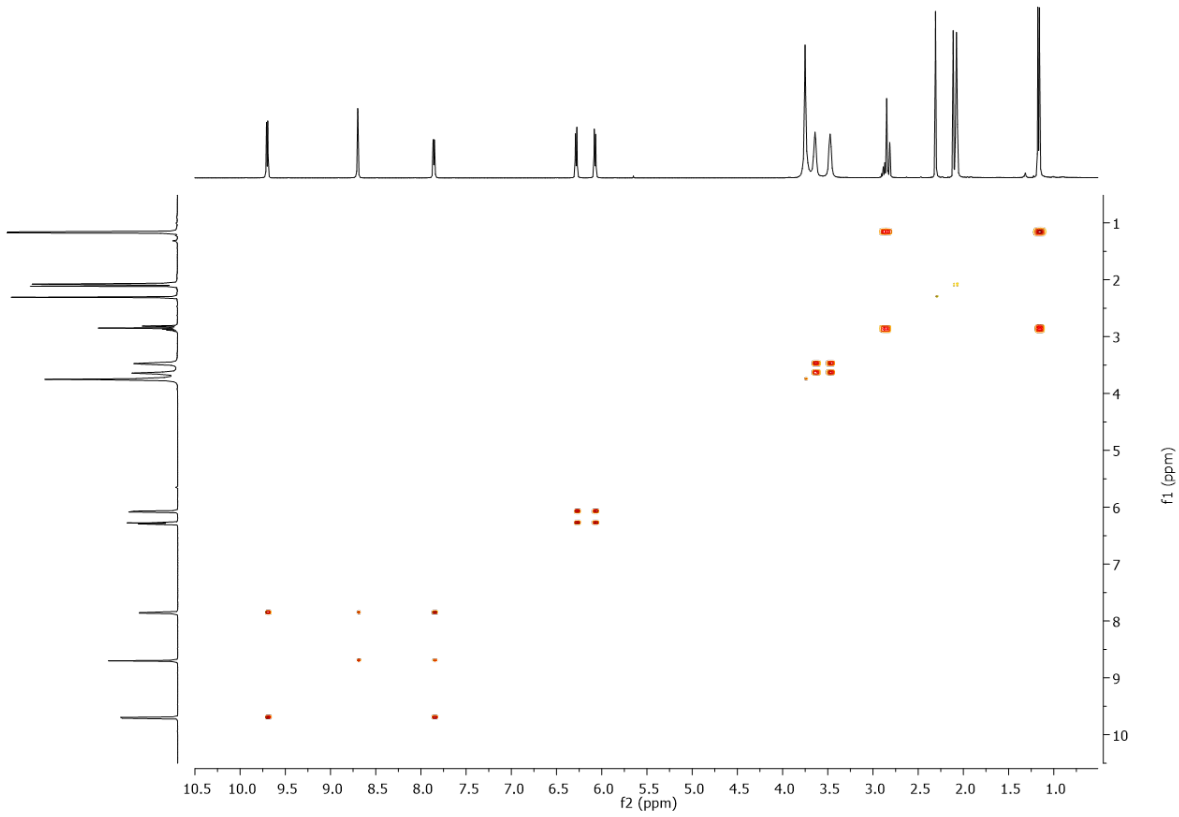

Figure S19. ^{1}H^{1}H-COSY spectrum of compound **Ru-pCy4** recorded in $(CD_3)_2CO$.

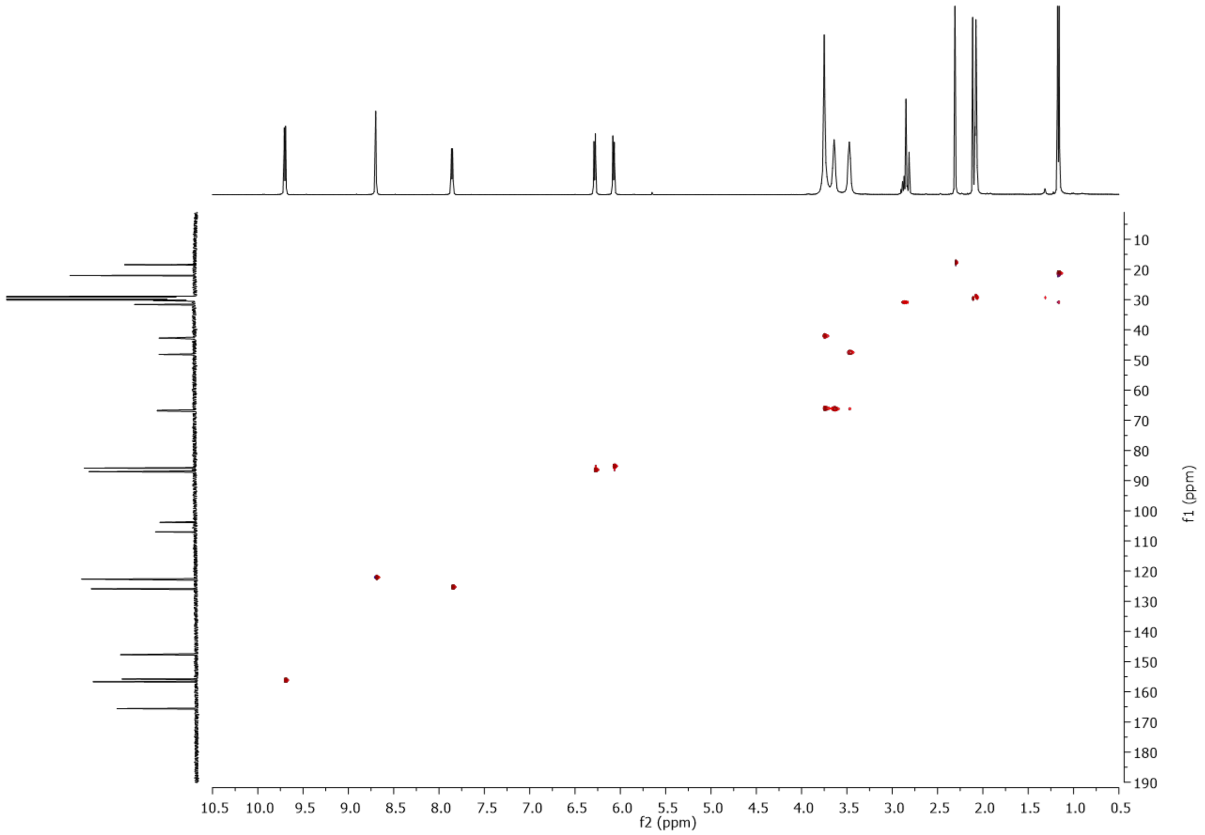

Figure S20. $^{1}H^{13}C$-HSQC spectrum of compound **Ru-pCy4** recorded in $(CD_3)_2CO$.

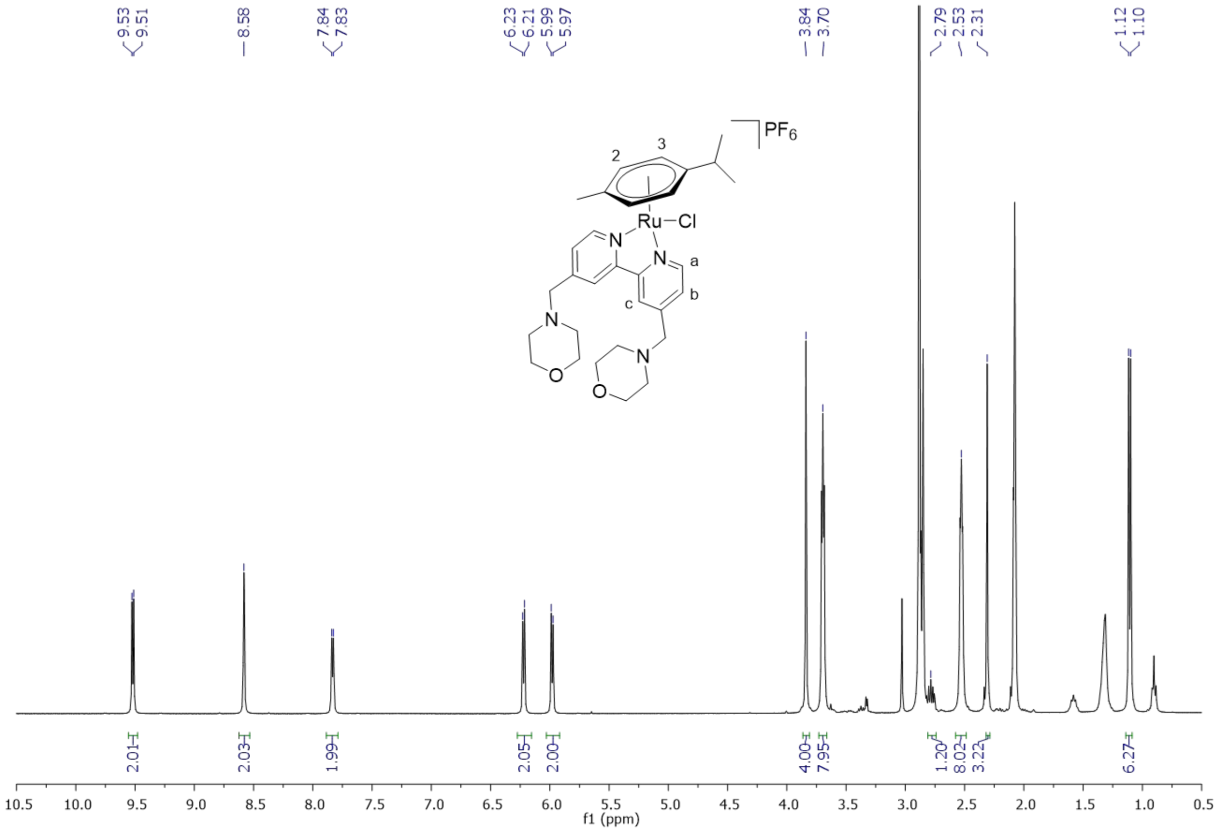

Figure S21. ^{1}H-NMR (400 MHz) spectrum of compound **Ru-pCy5** recorded in $(CD_3)_2CO$.

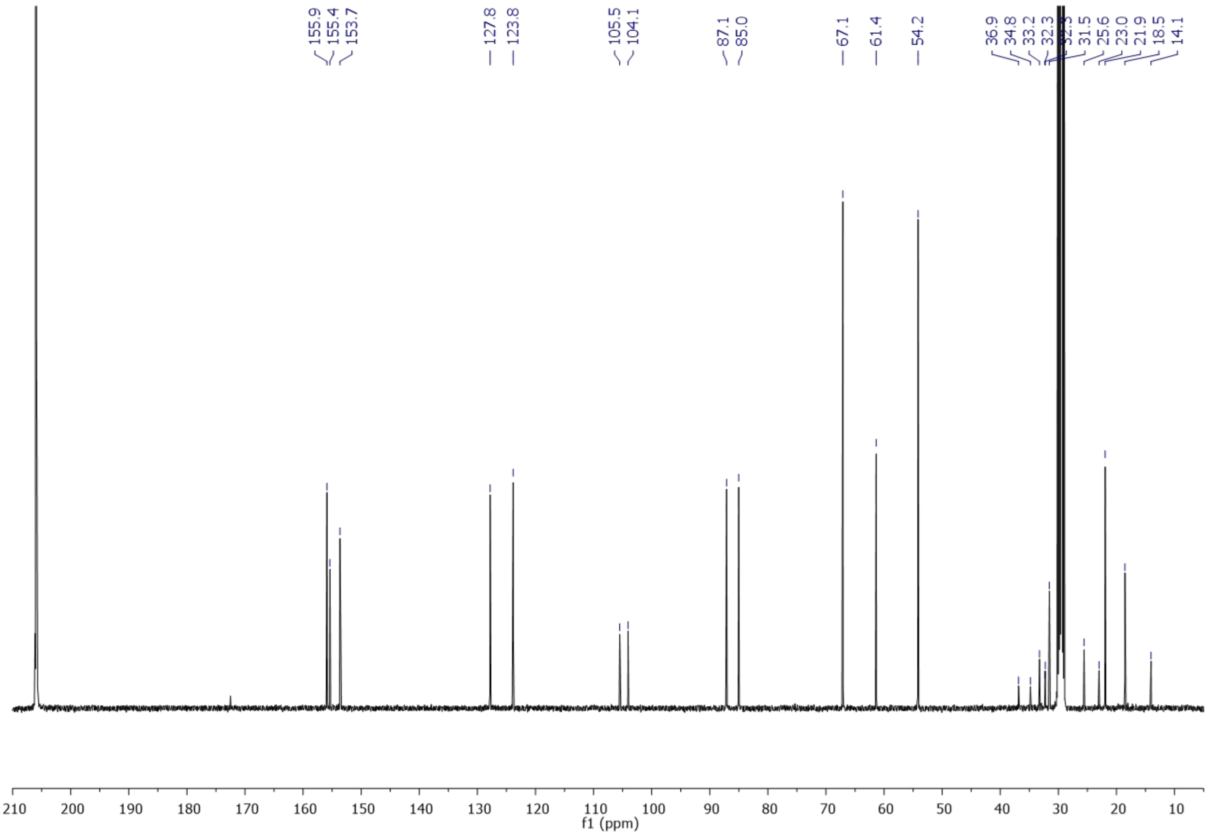

Figure S22. ^{1}C-NMR (100 MHz) spectrum of compound **Ru-pCy5** recorded in $(CD_3)_2CO$.

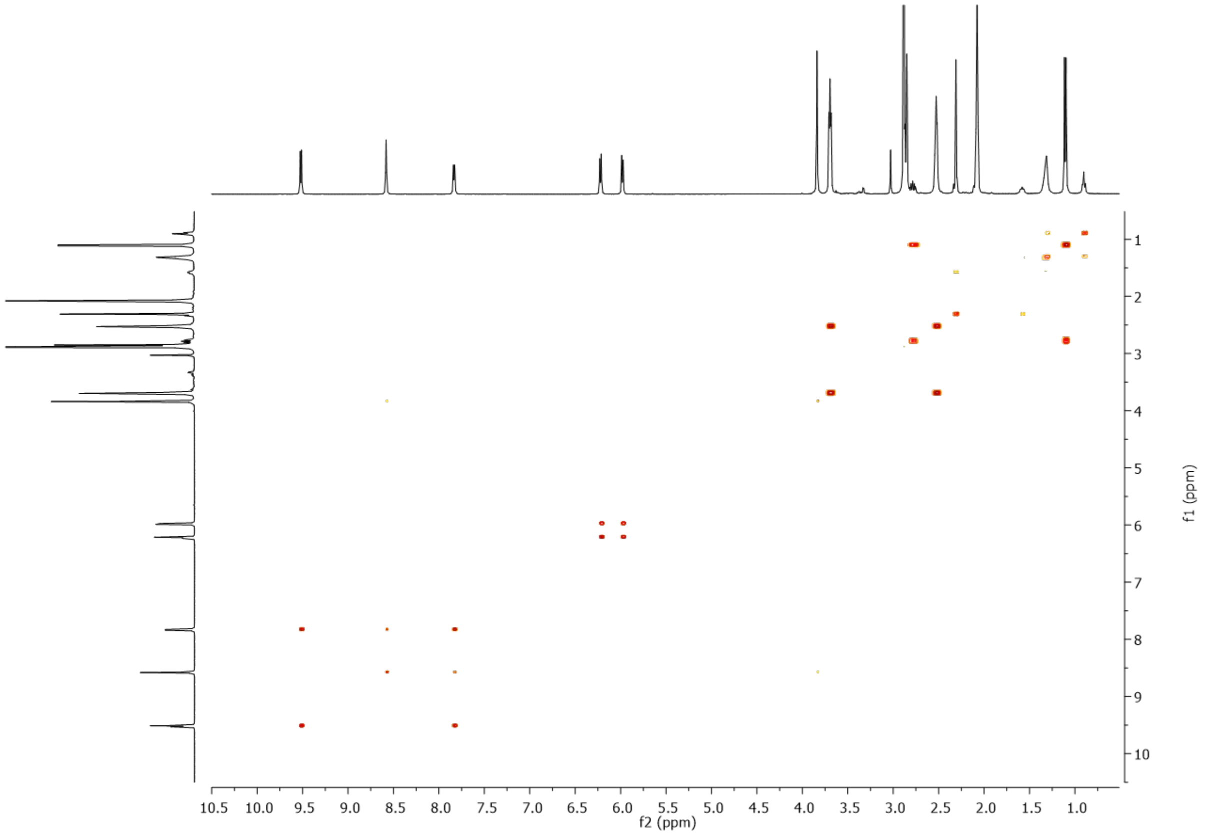

Figure S23. ^{1}H^{1}H-COSY spectrum of compound **Ru-pCy5** recorded in $(CD_3)_2CO$.

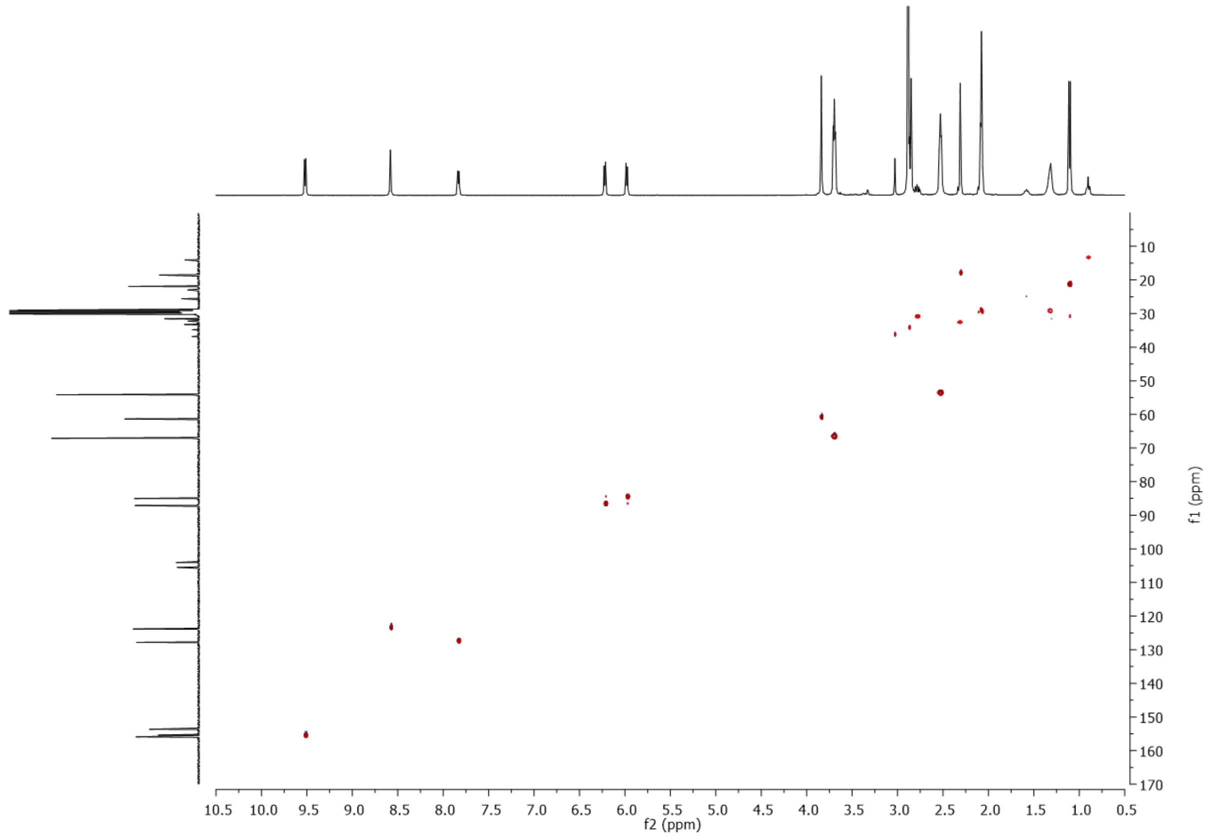

Figure S24. $^{1}H^{13}C$-HSQC spectrum of compound **Ru-pCy5** recorded in $(CD_3)_2CO$.

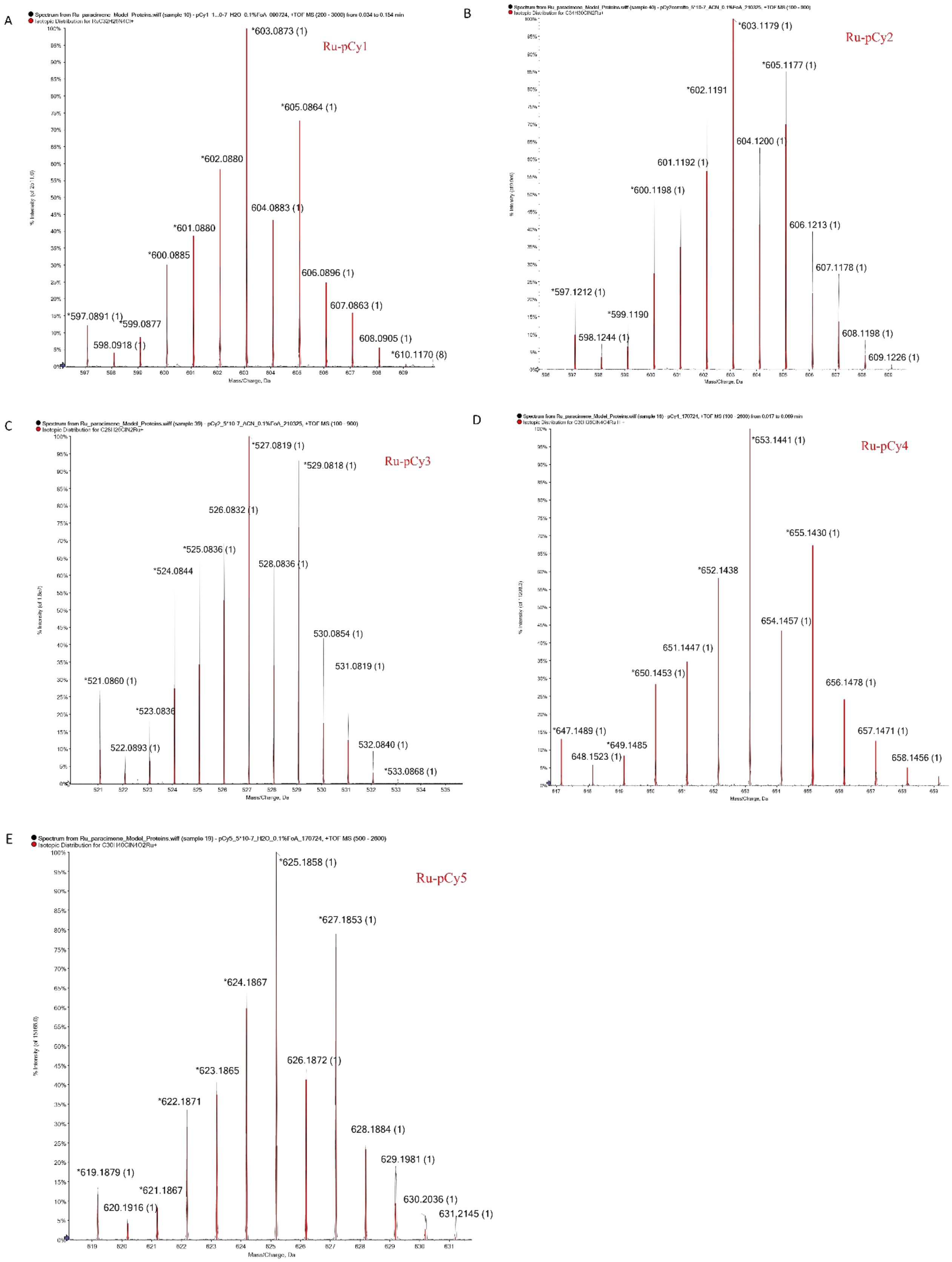

Figure S25. HR-ESI-MS of the **Ru-pCy1-5** complexes: A) **Ru-pCy1,** $[M]^+$ m/z = 603.08691 (theoretical for $C_{32}H_{26}ClN_4Ru$ $[M-PF_6]^+$: m/z =603.11794); B) **Ru-pCy2**, $[M]^+$ m/z = 603.08691 (theoretical for $C_{34}H_{30}ClN_2Ru$ $[M-PF_6]^+$: m/z =603.11355); C) **Ru-pCy3**, $[M]^+$ m/z = 527.08192 (theoretical for $C_{28}H_{26}ClN_2Ru$ $[M-PF_6]^+$: m/z =527.08225); D) **Ru-pCy4**, $[M]^+$ m/z = 653.14376 (theoretical for $C_{30}H_{36}ClN_4O_4Ru$ $[M-PF_6]^+$: m/z =653.14631); E) **Ru-pCy5**, $[M]^+$ m/z = 625.18585 (theoretical for $C_{30}H_{40}ClN_4O_2Ru$ $[M-PF_6]^+$: m/z =625.18778); in acetonitrile.

a)

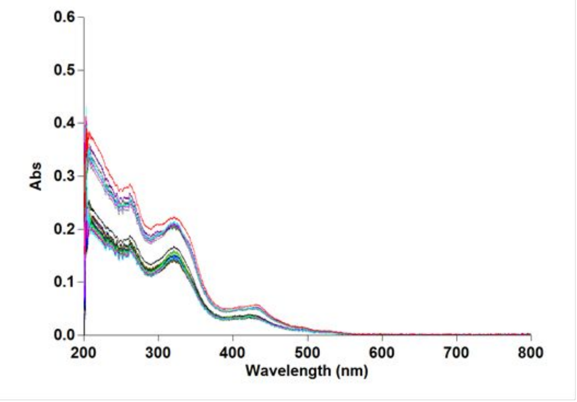

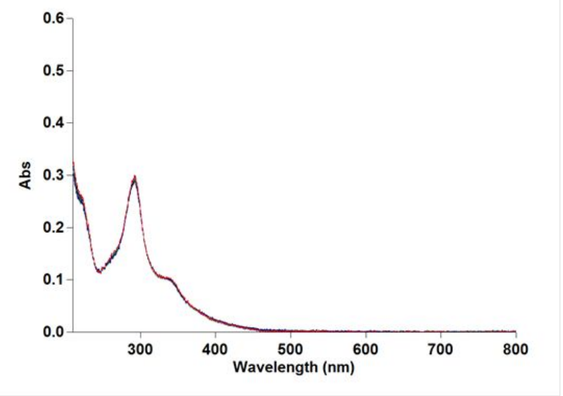

b)

c)

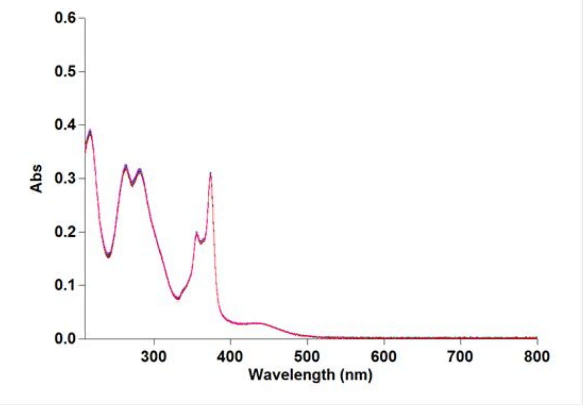

d)

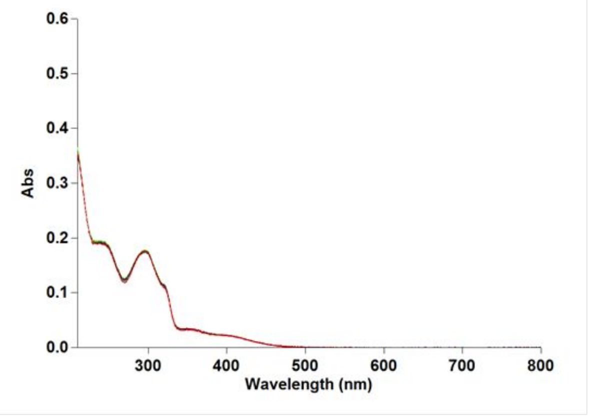

e)

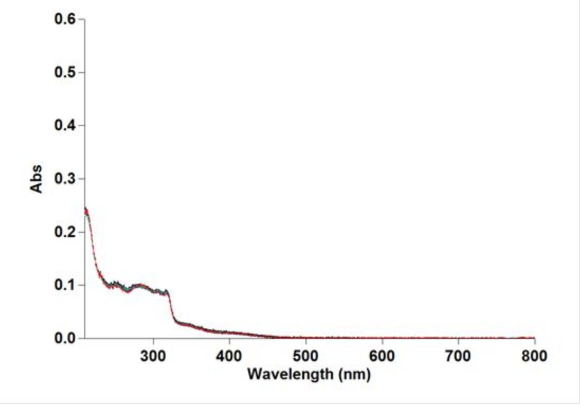

Figure S26. UV-vis spectra of ruthenium compounds dissolved in PBS buffer, pH 7.4 at final complex concentration of 10^{-5}M: a) **Ru-pCy1**; b) **Ru-pCy2**; c) **Ru-pCy3**; d) **Ru-pCy4**; e) **Ru-pCy5**.

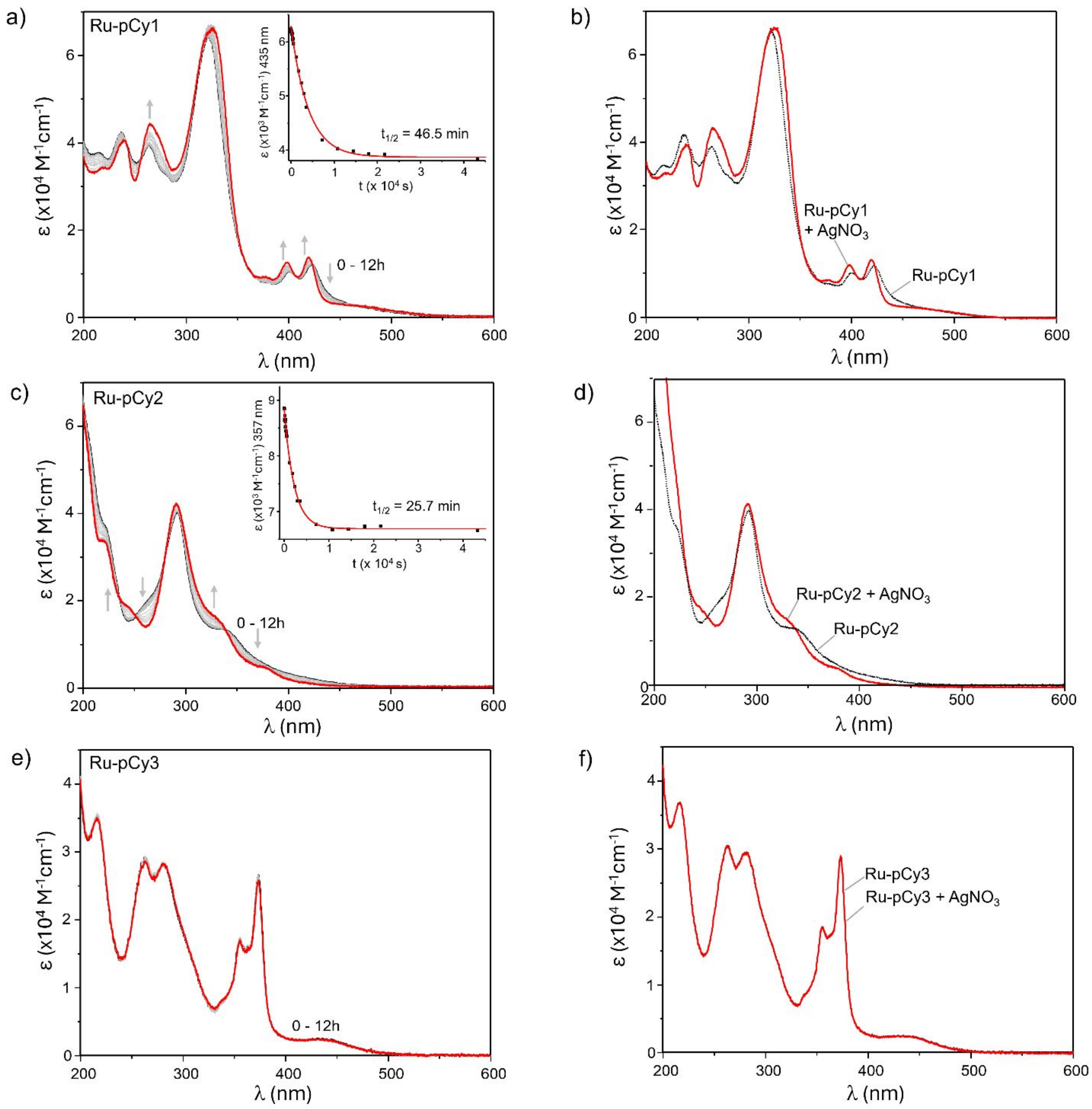

Figure S27. Ru-Cl hydrolysis for complexes **Ru-pCy1-2-3** as followed by UV-vis measurements in aqueous solutions at different times (up to 12 hours). For each complex, the inset on the left shows the variation in the MLCT region, while the figure on the right compares the spectra recorded in the absence and in the presence of 1 equiv. of $AgNO_3$, the latter providing an immediate visualization of the aquo-complex spectrum. **Ru-pCy1** before a) and after b) the addition of $AgNO_3$. **Ru-pCy2** before c) and after d) the addition of $AgNO_3$. **Ru-pCy3** before e) and after f) the addition of $AgNO_3$. No appreciable variations were observed for complex **Ru-pCy3** ([**Ru-pCy1-2-3**] = 10µM, pH 7, 298K).

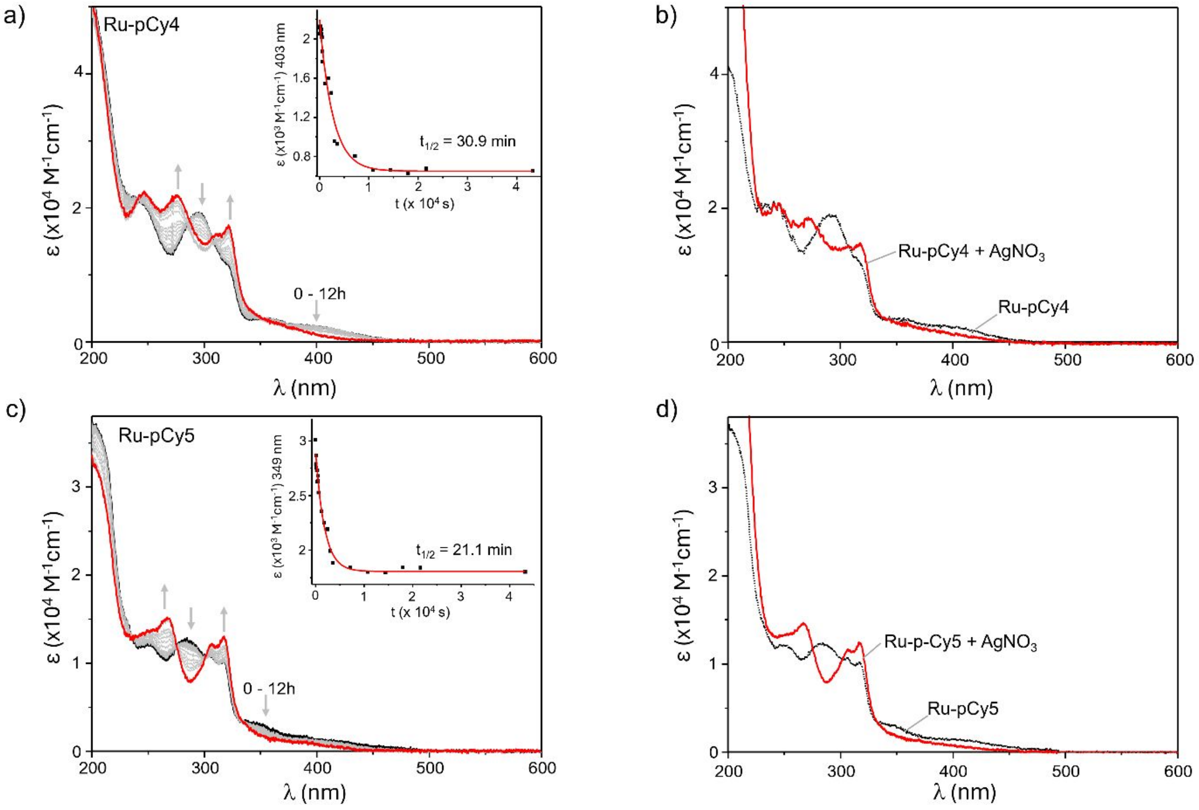

Figure S28. Ru-Cl hydrolysis for complexes **Ru-pCy4-5** as followed by UV-vis measurements in aqueous solutions at different times (up to 12 hours). For each complex, the inset on the left shows the variation in the MLCT region, while the figure on the right compares the spectra recorded in the absence and in the presence of 1 equiv. of $AgNO_3$, the latter providing an immediate visualization of the aquo-complex spectrum. **Ru-pCy4** before a) and after b) the addition of $AgNO_3$. **Ru-pCy5** before c) and after d) the addition of $AgNO_3$. ([**Ru-pCy4-5**] = 10μM, pH 7, 298K).

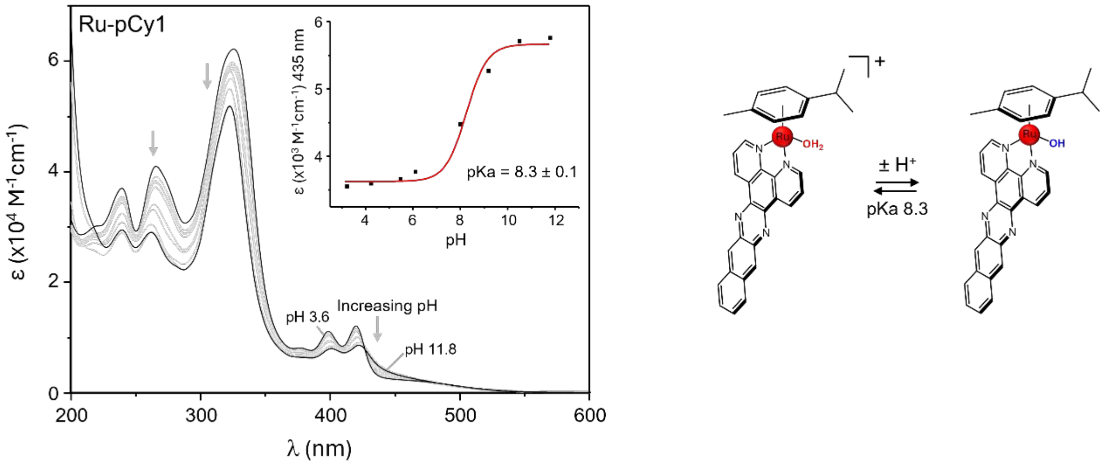

Figure S29. UV-vis spectra of aqueous solutions of **Ru-pCy1** at different pH values. The inset shows the variation of the absorbance at 435 nm with pH; the full line is the computational fit giving the pKa value reported. A schematic representation of the protonation equilibria between the charged $[(\eta^6\text{-p-Cymene})Ru(dppn)(H_2O)]^+$ and neutral $[(\eta^6\text{-p-Cymene})Ru(dppn)(OH)]$ species is provided on the right ([**Ru-pCy1**] = 10 μM, 298 K).

a)

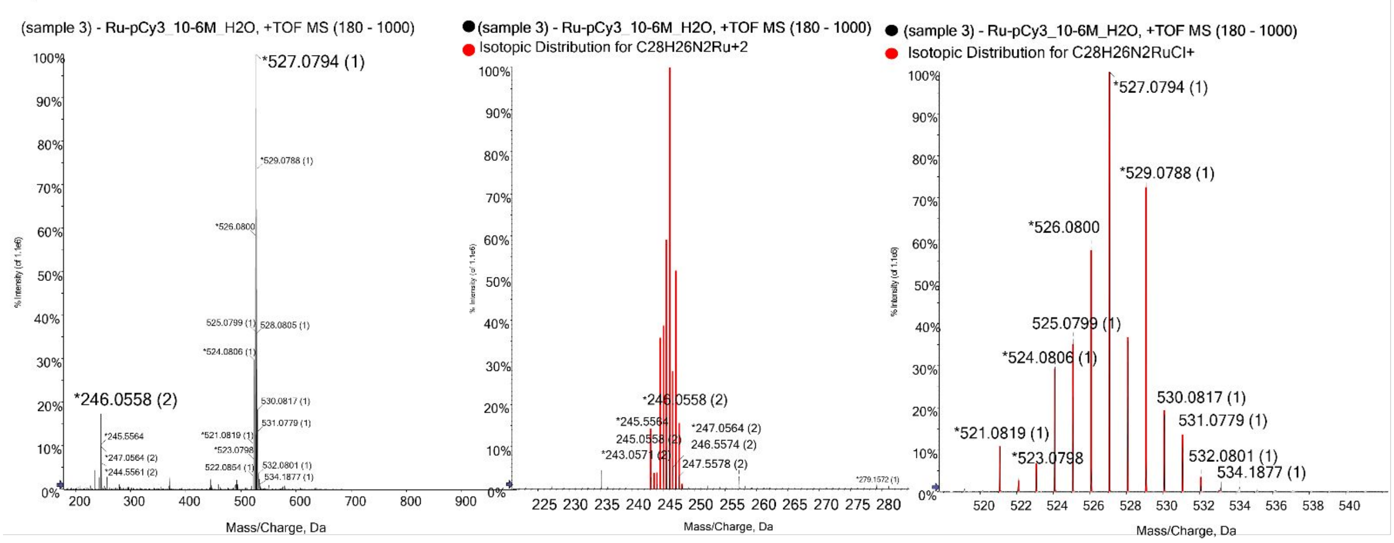

b)

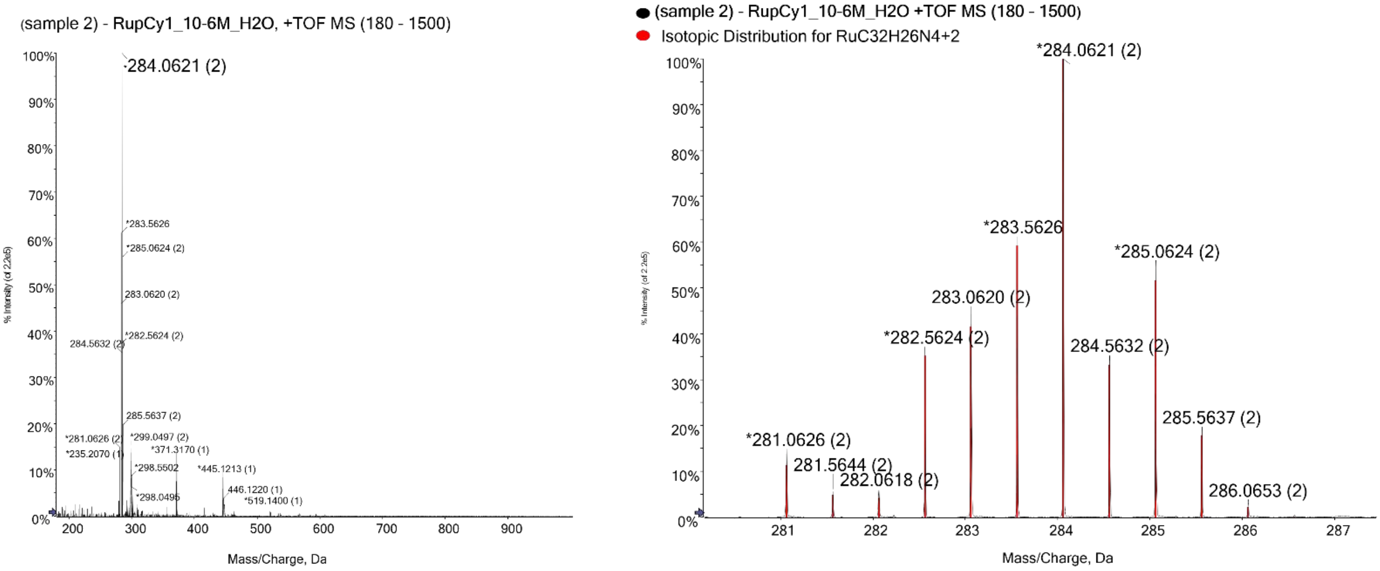

Figure S30. Superimposition of the theoretical and the experimental isotopic distribution of a) **Ru-pCy3,** HR-ESI-MS: $[M]^+$ m/z = 527.0794 (theoretical for $[RuC_{28}H_{26}ClN_2]^+$: m/z =527.08225), HR-ESI-MS: $[M]^{2+}$ m/z = 246.0558 (theoretical for [RuC28H26N2]$^+$: m/z =246.0564) and b) **Ru-pCy1,** HR-ESI-MS: $[M]^{2+}$ m/z = 284.0621 (theoretical for $C_{32}H_{26}N_4Ru]^{2+}$: m/z = 284.0595).

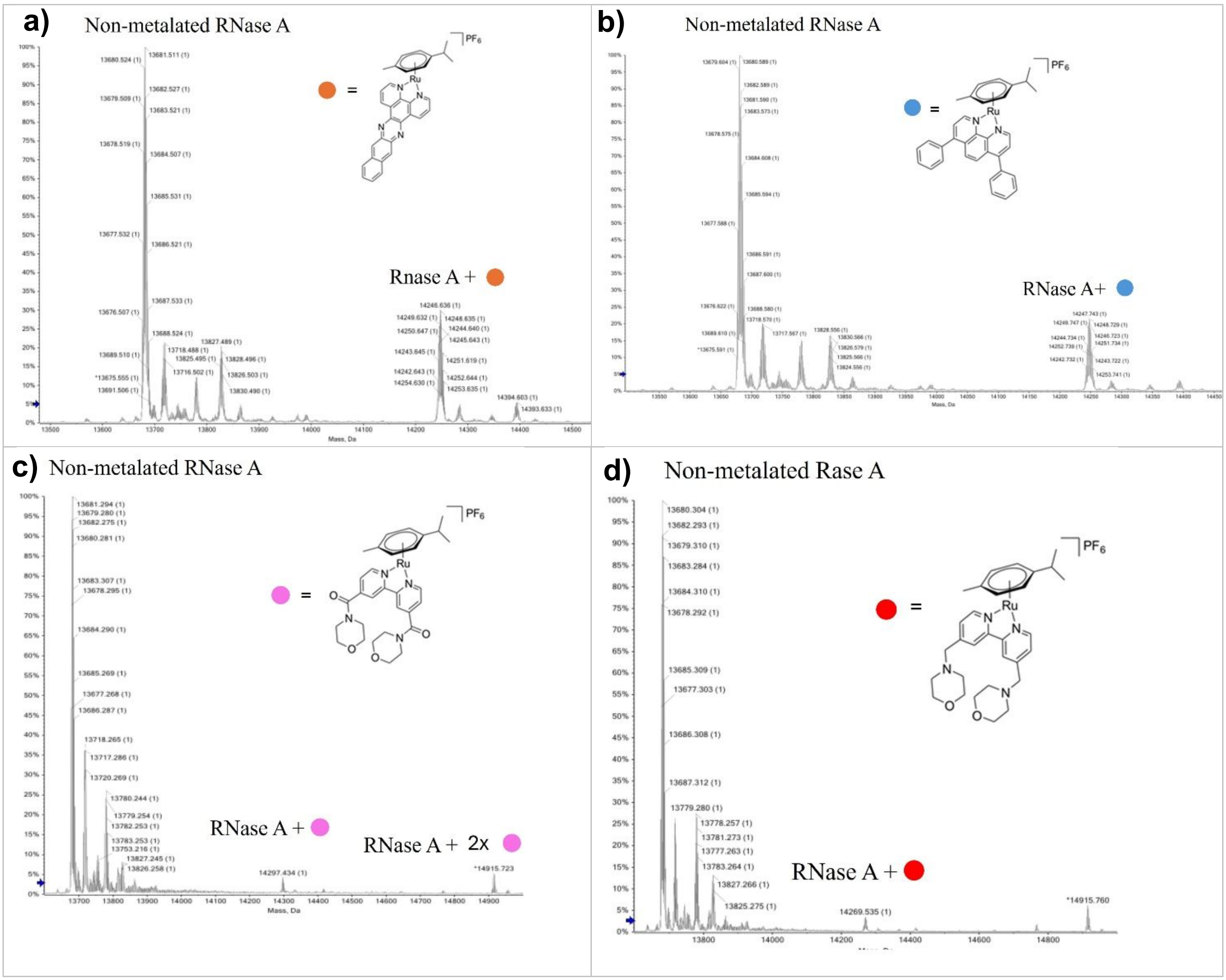

Figure S31. Deconvoluted ESI-Q-TOF spectra of RNaseA with four complexes in a 1:3 molecules to ruthenium ratio at 24 hours: a) **Ru-pCy1**; b) **Ru-pCy2**; c) **Ru-pCy4**; d) **Ru-pCy5**

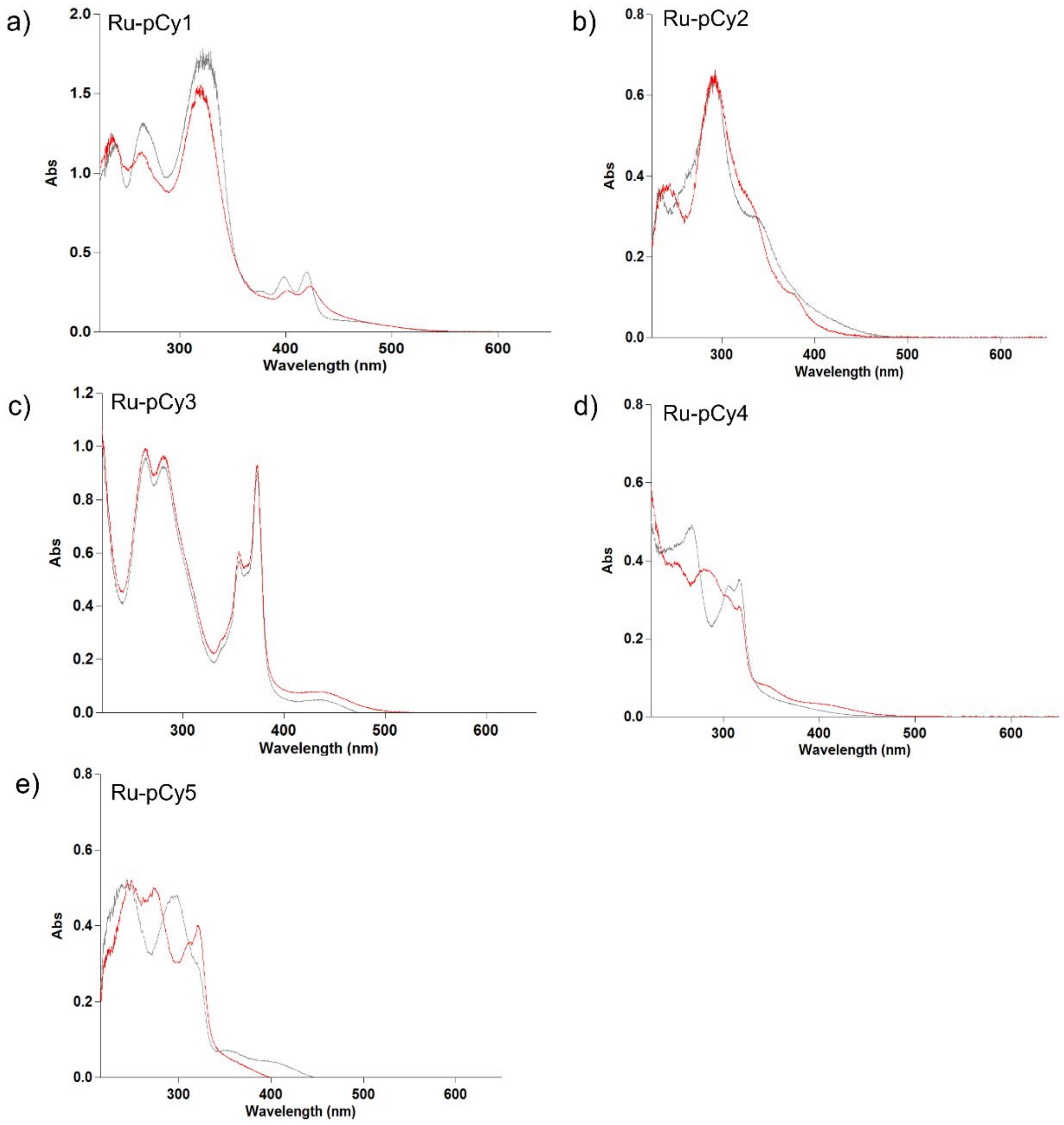

Figure S32. UV-vis spectra of aqueous solutions of ruthenium compounds and SOD (molar ratio 1:3 protein to metal) at final protein concentration of 10^{-5} M; a) **Ru-pCy1**; b) **Ru-pCy2**; c) **Ru-pCy3**; d) **Ru-pCy4**; e) **Ru-pCy5**.

Table S1. MIC values of tested compounds. Chloramphenicol is included as a reference antibiotic control.

**Strain**	**Ru-pCy 1**	**Ru-pCy2**	**Ru-pCy3**	**Ru-pCy4**	**Ru-pCy5**	**chloramphenicol**
B. subtilis 168	128 µg/ml	256 µg/ml	256 µg/ml	>256 µg/ml	>256 µg/ml	16 µg/ml
B. cenocepacia K56-2	256 µg/ml	>256 µg/ml	>256 µg/ml	256 µg/ml	256 µg/ml	64 µg/ml
B. cenocepacia J2315	128 µg/ml	256 µg/ml	256 µg /ml	256 µg/ml	256 µg/ml	32 µg/ml
